# Dietary tryptophan links encephalogenicity of autoreactive T cells with gut microbial ecology

**DOI:** 10.1038/s41467-019-12776-4

**Published:** 2019-10-25

**Authors:** Jana K. Sonner, Melanie Keil, Maren Falk-Paulsen, Neha Mishra, Ateequr Rehman, Magdalena Kramer, Katrin Deumelandt, Julian Röwe, Khwab Sanghvi, Lara Wolf, Anna von Landenberg, Hendrik Wolff, Richa Bharti, Iris Oezen, Tobias V. Lanz, Florian Wanke, Yilang Tang, Ines Brandao, Soumya R. Mohapatra, Lisa Epping, Alexandra Grill, Ralph Röth, Beate Niesler, Sven G. Meuth, Christiane A. Opitz, Jürgen G. Okun, Christoph Reinhardt, Florian C. Kurschus, Wolfgang Wick, Helge B. Bode, Philip Rosenstiel, Michael Platten

**Affiliations:** 1 DKTK Clinical Cooperation Unit Neuroimmunology and Brain Tumor Immunology, German Cancer Research Center (DKFZ), Heidelberg, Germany; 2 Department of Neurology, Medical Faculty Mannheim, Heidelberg University, Heidelberg, Germany; 3 Institute of Clinical Molecular Biology, University Kiel, Kiel, Germany; 4 Molecular Biotechnology, Department of Biosciences and Buchmann Institute for Molecular Life Sciences (BMLS), Goethe-University Frankfurt, Frankfurt, Germany; 5 University Medical Center of the Johannes Gutenberg-University Mainz, Institute for Molecular Medicine, Mainz, Germany; 6 Center for Thrombosis and Hemostasis (CTH), University Medical Center of the Johannes Gutenberg University Mainz, Mainz, Germany; 7 Junior Group Brain Cancer Metabolism, German Cancer Research Center (DKFZ), Heidelberg, Germany; 8 University Hospital Münster, Department of Neurology with Institute of Translational Neurology, 48149 Münster, Germany; 9 nCounter Core Facility, Institute of Human Genetics, University of Heidelberg, Heidelberg, Germany; 10 Department of Neurology and National Center of Tumor Diseases (NCT), University Hospital Heidelberg, Heidelberg, Germany; 11 CCU Children’s Hospital and Metabolic Center Heidelberg, Heidelberg, Germany; 12 DKTK Clinical Cooperation Unit Neurooncology, German Cancer Research Center (DKFZ), Heidelberg, Germany; 13 Present Address: Institute of Neuroimmunology and Multiple Sclerosis, Center for Molecular Neurobiology Hamburg, University Hospital Hamburg-Eppendorf, Hamburg, Germany; 14 Present Address: Immatics Biotechnologies GmbH, Tübingen, Germany; 15 Present Address: Division of Immunology and Rheumatology, Department of Medicine, Stanford University School of Medicine, Stanford, CA USA; 16 Present Address: Roche, Basel, Switzerland; 17 Present Address: Merck KGaA, Darmstadt, Germany; 18 Present Address: Department of Dermatology, Heidelberg University Hospital, Heidelberg, Germany

**Keywords:** Autoimmunity, Neuroimmunology, Microbiome, Multiple sclerosis, Nutrition

## Abstract

The interaction between the mammalian host and its resident gut microbiota is known to license adaptive immune responses. Nutritional constituents strongly influence composition and functional properties of the intestinal microbial communities. Here, we report that omission of a single essential amino acid - tryptophan – from the diet abrogates CNS autoimmunity in a mouse model of multiple sclerosis. Dietary tryptophan restriction results in impaired encephalitogenic T cell responses and is accompanied by a mild intestinal inflammatory response and a profound phenotypic shift of gut microbiota. Protective effects of dietary tryptophan restriction are abrogated in germ-free mice, but are independent of canonical host sensors of intracellular tryptophan metabolites. We conclude that dietary tryptophan restriction alters metabolic properties of gut microbiota, which in turn have an impact on encephalitogenic T cell responses. This link between gut microbiota, dietary tryptophan and adaptive immunity may help to develop therapeutic strategies for protection from autoimmune neuroinflammation.

## Abstract (web summary)

Food intake shapes intestinal microbiome composition, which in turn shapes adaptive immune responses. Here the authors show that dietary tryptophan restriction (DTR) protects mice from subsequent autoimmune neuropathology challenge by altering intestinal microbiota, highlighting the potential of diet-regulated microbiota to prevent immune pathology.

## Introduction

Impaired host–microbiota interaction in the gut may result in dysregulated systemic T cell responses contributing to autoimmune diseases in distant organs such as rheumatoid arthritis^1,2^ or autoimmune neuroinflammation^3–7^. Commensal intestinal bacteria control the differentiation of antigen-specific T cells into protective regulatory T cells (T_reg_)^8–10^ cells or pro-inflammatory T helper (T_H_) 17 cells^11,12^. Gut-associated lymphoid tissues are a major site for the phenotypic changes that allow the migration of autoreactive T cells to the brain^13^. The gut microbiota ecology is influenced by specific dietary constitutents such as salt, complex carbohydrates such as dietary fiber, or fermentation-derived short-chain fatty acids, which also have a known impact on organ autoimmunity^14–17^. Also, dietary protein restriction (DPR) has previously been shown to interfere with systemic immune responses^18^. Specifically, dietary tryptophan (trp) metabolites are crucial determinants of organ inflammation^18–20^. Trp metabolism plays a crucial role in regulating autoreactive immune responses^21–23^ and several pathways have been shown to sense and transmit immunomodulatory signals of trp and its metabolites. The stress kinase general control non-derepressible 2 (GCN2), for instance, is a central nutrient-sensing signaling hub that has been identified as a metabolic sensor of trp deprivation in response to indoleamine-2,3-dioxygenase 1 (IDO1)-mediated trp breakdown^24–26^. Additionally, a plethora of immunological effects of trp metabolism have been shown to be mediated by the aryl hydrocarbon receptor (AHR), a cytosolic transcription factor activated by mammalian and bacterial trp metabolites^27–33^. The relevance of dietary trp for regulating central nervous system (CNS) autoimmunity, however, remains largely unexplored.

Here we demonstrate that trp is a central dietary constituent, the omission of which blunts encephalitogenic T cell responses. Importantly, this effect is mediated by the gut microbiome rather than host Trp-sensing systems.

## Results

### Dietary trp is required for EAE induction

**Fig. 1:**
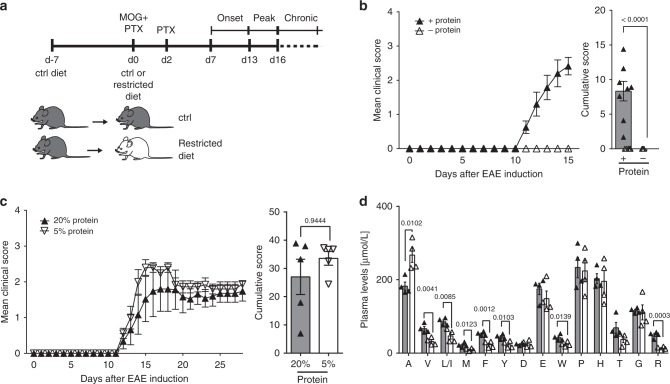
DPR prevents EAE induction. **a** Schematic representation of experimental procedure. **b** Mean clinical EAE scores and cumulative scores (+protein: *n* = 8, −protein: *n* = 10). **c** Mean clinical EAE scores and cumulative scores (20% protein: *n* = 5, 5% protein: *n* = 5). **d** Plasma amino acid concentrations of mice from **b** (*n* = 4). Statistics: Mann–Whitney *U*-test for **b**, **c**; unpaired two-tailed Student’s *t*-test for **d**. Each dot represents one individual mouse. Data are presented as mean ± SEM. Source data are provided as a Source Data file

We first determined the requirement of dietary protein for organ-specific immunity. When mice received a protein-free diet (Fig. 1a), clinical signs of EAE were abrogated (Fig. 1b). Of note, a diet with a reduced protein content (5% compared to 20% in the control diet) did not affect EAE disease severity (Fig. 1c). Amino acid profiling revealed that DPR induced a significant drop in plasma levels of valine, leucine/isoleucine, methionine, phenylalanine, tyrosine, trp, and arginine, with trp most profoundly reduced (Fig. 1d).

**Fig. 2:**
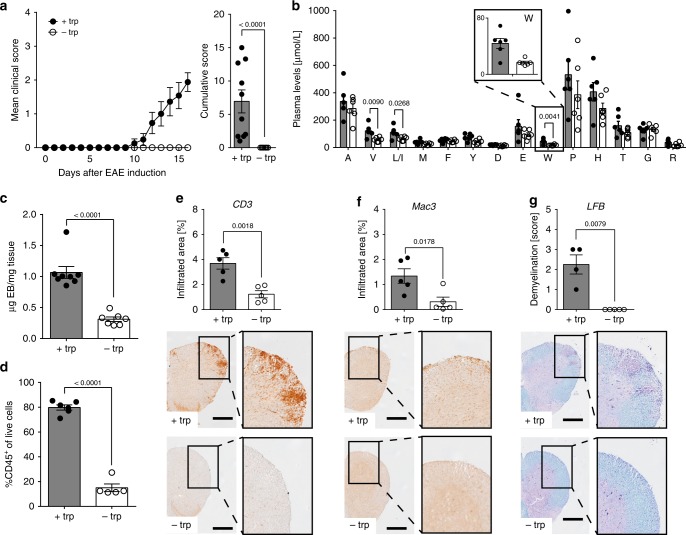
DTR inhibits EAE. **a** Mean clinical EAE scores and cumulative scores (+trp: *n* = 10; −trp, *n* = 10). **b** Plasma amino acid concentrations 16 days post-immunization (*n* = 6). **c** Blood–brain barrier (BBB) disruption in spinal cord as assessed by Evan’s Blue (EB) on d15 post-immunization (+trp: *n* = 8; −trp: *n* = 7). **d** Flow cytometric analysis of leukocyte infiltration into the spinal cord on d18 post-immunization (+trp: *n* = 5; −trp: *n* = 6). Displayed as CD45^+^ cells of live single cells. **e**–**g** Spinal cord sections of EAE mice were stained for **e** T cells (*n* = 5 vs. *n* = 5), **f** macrophages (*n* = 5 vs. *n* = 5), and **g** demyelination (*n* = 4 vs. *n* = 5). Scale bars: 250 μm. Statistics: Mann–Whitney *U*-test for **a**, **g** unpaired two-tailed Student’s *t*-test for **b**–**f**. Each dot represents one individual mouse. Data are presented as mean ± SEM. Source data are provided as a Source Data file

Omission of trp from the diet alone was sufficient to clinically mimic the protective effects of DPR (Fig. 2a). Although EAE disease severity was more pronounced in male mice, the protective effect of DTR was gender-independent (Supplementary Fig. 1a) and not generally attributable to essential amino acids as mice fed a methionine (met)-free diet developed disease (Supplementary Fig. 1b, c). Plasma amino acid profiling revealed that DTR induced a significant drop in trp plasma levels by 50–60% to approximately 20 µM 16 days post-immunization (Fig. 2b). DTR was accompanied by lack of key hallmarks of EAE, such as blood–brain barrier (BBB) disruption (Fig. 2c), leukocyte, T cell or macrophage infiltration, or demyelination (Fig. 2d–g, Supplementary Fig. 1d). While similar results were obtained when analyzing CNS tissue obtained from mice on DPR (Supplementary Fig. 1e–g), mice on a complete or met-free diet showed these pathological hallmarks of EAE (Supplementary Fig. 1h–j).

**Fig. 3:**
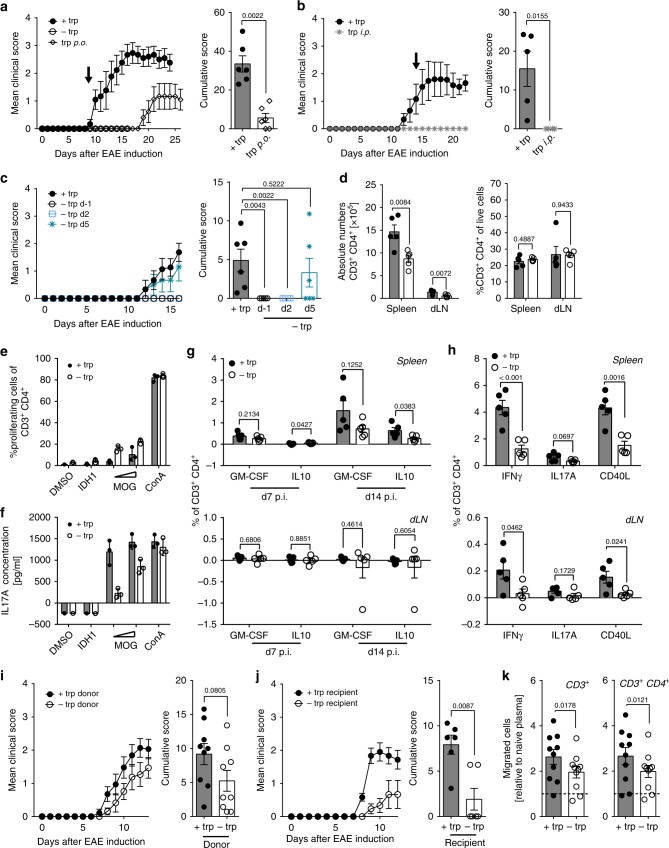
DTR interferes with T cell activation and migration. **a**–**c** Mean clinical EAE scores and cumulative scores in response to **a** oral trp supplementation started on d9 (+trp: *n* = 6; −trp: *n* = 5, trp p.o.: *n* = 6), **b** intraperitoneal trp supplementation initiated on d14 (+trp: *n* = 6; −trp i.p.: *n* = 6), or **c** time-dependent DTR (+trp: *n* = 6; −trp d−1: *n* = 5; −trp d2: *n* = 6; −trp d5: *n* = 6). **d** Secondary lymphoid organs were isolated from EAE mice on d13 (+trp: *n* = 6; −trp: *n* = 6). Left: Absolute CD3^+^ CD4^+^ T cell numbers. Right: Frequency of CD3^+^ CD4^+^ T cells of live cells. **e** Proliferation assay with T cells isolated from immunized mice on a trp-proficient or -deficient diet after stimulation with 1 or 10 µg/ml MOG_35–55_, 10 µg/ml isocitrate dehydrogenase 1_132–142_ (IDH1_124–142_) or 2 µg/ml Concanavalin A (ConA). **f** IL17A ELISA measurements after restimulation from **e**. **g** Secondary lymphoid organs were isolated from EAE mice on d7 or d14 (*n* = 5). After in vitro restimulation with MOG_35–55_ or IDH1_124–142_ secretion of IL-10 and GM-CSF was measured by ICS. **h** Secondary lymphoid organs were isolated from EAE mice on d13 (*n* = 5). After in vitro restimulation with MOG_35–55_ or PLP_139–151_ expression of INFγ, IL17A, and CD40L was measured by ICS. **i** Mean clinical EAE scores and cumulative scores after adoptive transfer of T cells (*n* = 9). **j** Mean clinical EAE scores and cumulative scores of ctrl and DTR mice after adoptive T cells primed in ctrl animals (*n* = 6). **k** MBMEC transmigration assay after pre-treatment of T cells with plasma (+trp: *n* = 10, −trp: *n* = 10; pooled from three independent experiments). Statistics: Mann–Whitney *U*-test for **a**–**c**, **i**, **j**; unpaired two-tailed Student’s *t*-test for **g**, **h**; paired two-tailed Student’s *t*-test for **k**. Each dot represents one individual mouse except for **e** and **f** where technical replicates are shown. Data are presented as mean ± SEM, or mean ± SD in **e** and **f**. Source data are provided as a Source Data file

We next tested whether the effect induced by DTR is dependent on the oral delivery route. Therefore, myelin oligodendrocyte glycoprotein (MOG)-immunized mice, which received a trp-free diet for 9 days and did not show any clinical EAE signs, were reconstituted with trp orally (p.o.). These mice developed clinical signs with a time lag of 10 days (Fig. 3a). In contrast, systemic supplementation via daily intraperitoneal injections (i.p.) did not rescue the phenotype of DTR (Fig. 3b). These findings indicate that indeed presence of luminally delivered trp is required for the induction of autoimmune neuroinflammation. Of note, corticosteroid levels were not significantly elevated in plasma of mice that received a trp-free diet (Supplementary Fig. 2a), indicating that abrogation of autoimmune neuroinflammation is not a consequence of an unspecific stress response to DTR. Interestingly, plasma leptin levels were drastically reduced in DTR mice (Supplementary Fig. 2b), while active ghrelin was hardly detectable in plasma from DTR and control mice (Supplementary Fig. 2c), which argues against a strong systemic fasting response.

We next addressed the question whether dietary trp is required for the activation and priming of antigen-specific T cells. DTR in the early induction phase was critical for protection from neuroinflammation, as mice on DTR initiated on day 5 post-immunization developed EAE, while DTR on day 2 post-immunization prevented disease (Fig. 3c). DTR started on the day of immunization reduced absolute CD4^+^ T cell counts, but not relative frequency within all CD45^+^ cells, both in spleen and draining lymph nodes (dLN) (Fig. 3d, Supplementary Fig. 3a). However, MOG-reactive CD4^+^ T cell responses were still mounted in the absence of dietary trp, as shown by ex vivo proliferation assays (Fig. 3e, Supplementary Fig. 3b). Also, adoptively transferred T cell receptor (TCR)-transgenic MOG_35–55_-specific T cells from 2D2 mice were not impaired in their ability to proliferate within the circulation of recipient mice on DTR (Supplementary Fig. 3c), indicating that DTR does not generally suppress the induction or maintenance of autoreactive T cells. However, in response to DTR we detected a loss of IL17A, but also a reduced IL-10 secretion after ex vivo peptide restimulation (Fig. 3f, g). A phenotypic shift of MOG-reactive T cells towards a reduction of IFNγ-secreting CD40L^+^ T_H_ cells in both spleen and dLN in DTR mice was already observed during the induction phase (Fig. 3h, Supplementary Fig. 3d, e). In line with decreased expression of IL17A, IFNγ and CD40L in response to peptide restimulation, we found the frequency of MOG tetramer-reactive CD4^+^ T cells to be decreased during the induction phase before disease onset with an enrichment of tetramer-positive cells in the CD44^high^ effector fraction (Supplementary Fig. 3f). Of note, T_reg_ frequencies and anti-MOG serum auto-antibody titers were not affected in DTR mice (Supplementary Fig. 4a, b). Upon adoptive transfer of MOG_35–55_-specific T cells, DTR led to a reduction of CXCR3 expression, a chemokine receptor involved in regulating migratory properties of T cells^34^, while CCR6 expression was not affected (Supplementary Fig. 4c). Nanostring-based transcriptional profiling of peripheral MOG-reactive T cells revealed increased expression of *Il2ra*, *Il2rb*, *Nptn* (Neuroplastin), and *Furin* in response to DTR (Supplementary Fig. 4d–g, Supplementary Data 1). Collectively, these data indicate that DTR induces a distinct phenotypic change in systemic autoreactive T cells that prevents formation of encephalitogenic T cells.

To test the impact of DTR on the function of primed MOG-specific T cells in more detail, ex vivo T_H_17-polarized MOG-reactive CD4^+^ T cells from mice on DTR or complete diet were transferred into recipient mice that all received a control diet. MOG-reactive T cells transferred from mice on DTR were fully capable to induce neuroinflammation after ex vivo stimulation (Fig. 3i, Supplementary Fig. 4h–j). In contrast, EAE was blunted after adoptive transfer of MOG-specific CD4^+^ T cells primed in mice on a complete diet into recipient DTR mice (Fig. 3j). Interestingly, pre-treatment of activated T cells with plasma of DTR mice resulted in impaired transmigration towards an ex vivo BBB modeled by murine brain microvascular endothelial cells (MBMEC; Fig. 3k), suggesting that a soluble factor in DTR mice interferes with T cell migration into the CNS.

These data suggest that dietary trp is dispensable for the priming of MOG-reactive T cells, but DTR exerts its impact by both reducing the number of circulating MOG_35–55_-reactive activated CD4^+^ T cells and by altering the phenotype as well as their migratory properties. This effect is reversible and driven by the continuous presence of an environmental variable.

### DTR mediates disease protection independent of GCN2 and AHR

**Fig. 4:**
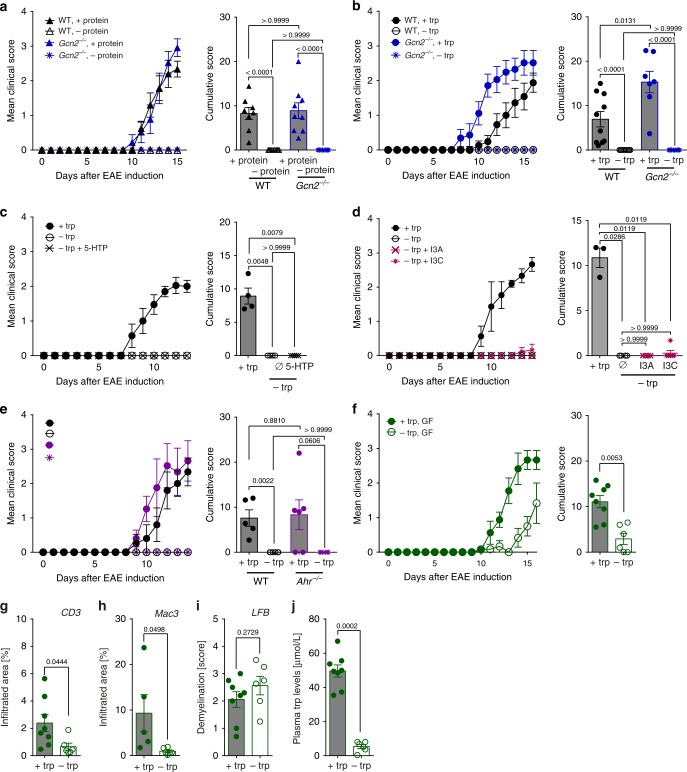
EAE inhibition driven by DPR and DTR is independent of GCN2 and AHR. **a** Mean clinical EAE scores and cumulative scores (WT, +protein: *n* = 8; WT, −protein: *n* = 10; *Gcn2*^*−/*^^−^,  +protein: *n* = 9; *Gcn2*^*−/*^^*−*^, −protein: *n* = 10). **b** Mean clinical EAE scores and cumulative scores (WT, +trp: *n* = 10; WT, −trp: *n* = 10; *Gcn2*^*−/*−^ , +trp: *n* = 7; *Gcn2*^*−/*−^, −trp: *n* = 10). **c** Mean clinical EAE scores and cumulative scores after 5-hydroxytryptophan (5-HTP) supplementation (+trp: *n* = 4; −trp: *n* = 6, −trp+ 5-HTP: *n* = 5). **d** Mean clinical EAE scores and cumulative scores of mice supplemented with indole-3-aldehyde (I3A) or indole-3-carbinole (I3C) (+trp: *n* = 3; −trp: *n* = 3; −trp +  I3A: *n* = 6; −trp + I3C: *n* = 6). **e** Mean clinical EAE scores and cumulative scores (WT, +trp: *n* = 5; WT, −trp: *n* = 5; Ahr^*−/*^^−^,+trp: *n* = 5; Ahr^−/−^, −trp: *n* = 5). **f** Mean clinical EAE scores and cumulative scores of germ-free (GF) mice (+trp: *n* = 8; −trp: *n* = 6). **g** T cell infiltration (*n* = 8 vs. *n* = 5), **h** macrophage infiltration (*n* = 5 vs. *n* = 6), **i** demyelination (*n* = 8 vs. *n* = 6) and **j** plasma trp levels (*n* = 8 vs. *n* = 6) of mice from **f** were determined at d16 post-immunization. Statistics: Mann–Whitney *U*-test for **a**–**f**; unpaired two-tailed Student’s *t*-test for **g**–**j**. Each dot represents one individual mouse. Data are presented as mean ± SEM. Source data are provided as a Source Data file

In order to examine whether protection from EAE requires GCN2 activation as a result of dietary trp or protein deprivation, EAE was induced in GCN2*-*deficient (*Gcn2*^−/−^) mice. Surprisingly, *Gcn2*^*−/−*^ mice were equally resistant to EAE as WT mice when fed a protein-free (Fig. 4a, Supplementary Fig. 5a–c) or trp-free diet (Fig. 4b, Supplementary Fig. 5d–f). These data demonstrate that host GCN2 is dispensable for the protection of mice from EAE by DTR. In order for GCN2 to serve as a molecular sensor for trp depletion, a drop of trp levels from 50 µM to below 1 µM is required^24^. In line with these observations, we found GCN2 to be activated in T cells at trp concentrations of 0.25 µM, as measured by increased expression of the transcription factor C/EBP-homologous protein (CHOP) (Supplementary Fig. 5g). When analyzing spinal cord tissue, we found that DTR had no effect on the trp levels within the CNS (Supplementary Fig. 5h), indicating that trp levels are maintained in the CNS—at least for the period applied in our study protocol—despite omission of this essential amino acid from the diet.

Omission of dietary trp supply may affect trp metabolism via the 5-hydroxytryptamin (5-HT)/serotonin pathway. Besides being a classical neurotransmitter, serotonin has been implicated to regulate T cell activation and proliferation^35^. To evaluate whether the resistance to EAE induced by DTR could result from impaired serotonin signaling, we measured systemic serotonin concentrations in the plasma, which were not consistently altered in DTR mice (Supplementary Fig. 5i). To exclude the possibility that a functional limitation of serotonin drives the protective effect of DTR, mice were reconstituted with the active serotonin precursor 5-hydroxytryptophan (5-HTP). However, 5-HTP rescue did not reverse the phenotype induced by DTR (Fig. 4c). Also, neither supplementation of DTR mice with indole-3-aldehyde (I3A) nor indole-3-carbinole (I3C), activating ligands of the AHR^9,19,36^, nor genetic *Ahr* ablation were able to break resistance of DTR mice to EAE (Fig. 4d, e), indicating that host AHR signaling is not involved as sensing mechanism for the observed DTR effect.

To test whether resistance to EAE by DTR diet is dependent on the gut microbiome, we next tested the experimental setup in germ-free (GF) mice. Although it has previously been shown that the commensal gut flora is a prerequisite for triggering CNS autoimmunity in a spontaneous EAE model^5^, both GF DTR and control mice were susceptible to EAE induction (Fig. 4f), yet, the cumulative score of DTR mice was still significantly lower than in control animals, arguing for a partial microbiota-dependent effect. Although infiltration of T cells and macrophages was lower in GF mice that were fed a trp-free diet (Fig. 4g, h), demyelination was equally pronounced in both groups (Fig. 4i) at the peak of disease. In line with previous findings in specific-pathogen free (SPF) conditions, trp plasma levels were significantly reduced in response to DTR in GF mice (Fig. 4j). Interestingly, under GF conditions trp plasma levels dropped to as low as 5% of the value in control animals, whereas in SPF animals they were only reduced to 40% (Supplementary Fig. 5j), which may be due to the fact that bacteria capable of de novo trp synthesis are absent under GF conditions. In summary, these data demonstrate that the presence of gut microbiota plays a decisive role in preventing CNS infiltration of encephalitogenic T cells in response to DTR and that this trp-dependent effect is mediated by a yet unknown mechanism.

### DTR shapes colonic gut microbiota composition

**Fig. 5:**
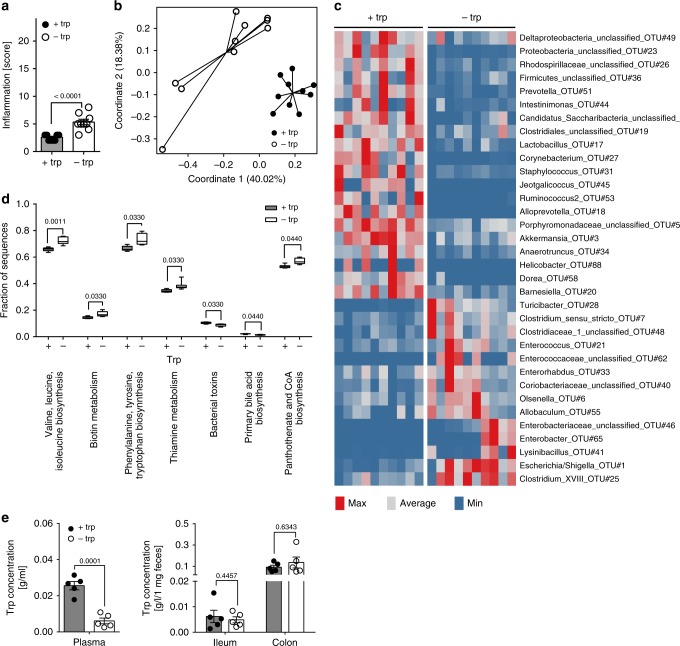
DTR alters gut microbiota composition. **a** Histopathological gut inflammation score of EAE mice on d14 post-immunization (+trp: *n* = 10; −trp: *n* = 10). **b**–**d** Profiling of microbial communities by 16S rRNA gene sequencing for mice from **a**. **b** Principal coordinate analysis (PCoA; Bray-Curtis) on colonic microbial communities. **c** Relative abundance of significant bacterial indicator genera depicted by a heatmap. **d** Predicted functional categories as determined by PICRUSt. **e** Analysis of trp levels in plasma, feces, and ileal contents of EAE mice on d13 post-immunization (+trp: *n* = 5; −trp: *n* = 5). Statistics: Mann–Whitney*U*-test for **a**–**d**; unpaired two-tailed Student’s *t*-test in **e**. In **d** the Mann–Whitney*U*-test was performed for pair-wise comparisons with the control group and *p* values were corrected according to Benjamini–Hochberg. Each dot represents one individual mouse. Data are presented as mean ± SEM. Source data are provided as a Source Data file or in Supplementary Data 2–4

Trp malnutrition has been shown to alter microbial ecology and drive intestinal inflammation^22^. Indeed, we could confirm that DTR induced a mild inflammation in the colon characterized by increased invasion of leukocytes and elevated accumulation of lymph follicles (Fig. 5a, Supplementary Fig. 6a). Mucosal immune cell infiltrates were dominated by macrophages, B cells, and CD4^+^ T cells in both groups with contraction of the B cell compartment in DTR mice (Supplementary Fig. 6b).

We next analyzed gut microbial community composition in response to DTR using 16S rRNA gene sequencing of feces obtained from MOG_35-55_-immunized mice in order to identify changes of the gut microbiota which could be responsible for the immune-modulatory effect. Microbial α-diversity (non-parametric Shannon index; a measure of species diversity within a given sample) was significantly reduced in the DTR vs. control group (Supplementary Fig. 6c) and β-diversity analysis (based on Bray–Curtis distances as a measure of diversity between samples) showed distinct clustering of the experimental groups in the respective principal coordinate analysis (PCoA; Fig. 5b). On the phylum level, a contraction of bacterial taxa belonging to the phyla Bacteroidetes and Verrumicrobia was detected in response to DTR, while the phyla Actinobacteria, Proteobacteria, and Firmicutes were increased in relative abundance (Supplementary Fig. 6d, Supplementary Data 2). Using indicator analysis of the DTR vs. the control group on the level of genera, we further detailed taxonomical shifts induced in response to DTR and found *Akkermansia*, *Lactobacillus*, and *Barnesiella* to be decreased (Fig. 5c, Supplementary Data 3). From predictive functional profiling of microbial communities (PICRUSt; phylogenetic investigation of communities by reconstruction of unobserved states)^37^ we can infer that DTR induces alterations in the gut microbial phylogeny, which may subsequently affect key metabolic pathways such as amino acid synthesis (Fig. 5d, Supplementary Data 4). In line with the prediction that DTR drives accumulation of microbial species that synthesize trp de novo, we confirmed that indeed there is no significant drop of luminal trp levels in either ileum or colon, whereas plasma concentrations were strongly influenced (Fig. 5e). Systemic absorption of trp normally is a function of the proximal small intestine, thus luminal levels of trp in the lower parts of the intestine may well be uncoupled from serum levels.

Of note, gut bacteria may synthesize both the L- and D-form of trp. D-trp produced by Lactobacilli has recently been shown to inhibit inflammatory responses in an experimental model of allergic airway disease^38^. To exclude a shift of the L/D-trp ratio in response to DTR, which could be responsible for its immunomodulatory effects, we measured D-trp levels by chiral HPLC. Yet, neither plasma nor fecal D-trp concentrations were increased in response to DTR under the experimental conditions (Supplementary Fig. 6e, f).

Taken together, these results demonstrate that DTR has a profound impact on the gut microbiota composition, which subsequently affects metabolic pathways such as amino acid synthesis.

### DTR drives transcriptional alterations in intestinal mucosa

**Fig. 6:**
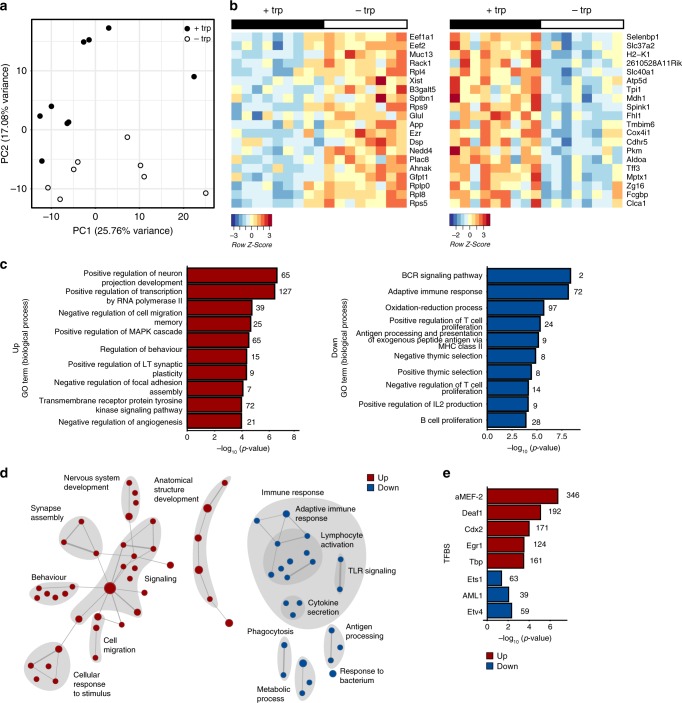
DTR regulates key molecular signaling pathways in colon mucosa. **a**–**e** Total colon tissue (distal) of EAE mice from Fig. 5 was isolated on d14 for RNA sequencing (+trp: *n* = 9; −trp: *n* = 9). **a** Principal component analysis (PCA) for RNA sequencing samples. **b** Heatmap of top 20 DEGs (up and down) in pair-wise comparisons between –trp and +trp. **c** Top 10 pathways (biological processes) of Gene Ontology (GO) enrichment analysis. **d** Enrichment map of regulated biological process GO terms. Each dot represents a significantly deregulated GO process, lines connect processes according to similarity of involved transcriptional modules. **e** TFBS analysis shows the enrichment of conserved transcription factor-binding sites in up- and downregulated transcripts. Source data are provided in Supplementary Data 5–9

To depict mucosal transcriptomal signatures associated with DTR, we performed RNA sequencing (RNA-seq) from colon mucosa 14 days post-immunization and identified 1237 and 1260 DEGs that were up- and downregulated in response to DTR, respectively (Fig. 6a, b, Supplementary Data 5). Formal gene set enrichment analysis using gene ontology^39^ revealed a striking downregulation of transcripts involved in regulation of both innate and adaptive immune responses, as well as antigen processing, while key processes in neuronal development and signaling were upregulated in response to DTR (Fig. 6c, d, Supplementary Data 6 and 7), indicating that DTR acts on activation of immune cells. To identify immunoregulatory pathways, we analyzed conserved transcription factor-binding sites (TFBS) in the regulated transcript sets. Binding sites for transcription factors aMEF-2, Deaf1, Cdx2, Egr1, and Tbp were among the top overrepresented sites in the upregulated genes, while sites for Etv4, AML1/Runx1, and Ets1 were overrepresented in the downregulated genes (Fig. 6e, Supplementary Data 8 and 9). Analysis of TFBS shows an enrichment of B cell-intrinsic factors (MEF2 (ref. ^40^), Deaf1 (ref. ^41^)), but also suggests T cell regulation processes, e.g. downregulation of Ets1 (ref. ^42^) and Runx1 (ref. ^43^) activity.

In summary, the clear signature of pathways involved in neuronal signaling and development hints towards a potential role of dietary trp in regulating the gut–brain axis. Although the results do not directly suggest an overt mechanism how the crosstalk between gut microbiota and the host's immune system mediate the protective effects of DTR on EAE development, the data show a profound effect on local immune responses in the gut mucosa and provide a starting point for future studies.

## Discussion

Here we demonstrate that omission of the essential amino acid trp from the diet is sufficient to inhibit encephalitogenic T cell responses and prevent CNS autoimmunity in a murine MS model. Although we did not formally test all essential amino acids, in this setting methionine restriction was not able to modify the clinical course of EAE, arguing against a general effect of essential AA deprivation. Together with other studies where trp deprivation phenocopied the strong immunoregulatory effects of DPR^18,22^, these data highlight the critical role of trp metabolism in immunosuppression^19,21,44^ and suggest that dietary trp is specifically required for the induction of CNS autoimmunity. The question arises as to what is the exact mechanism of DTR-induced immunomodulation.

In our experiments, deprivation of trp was associated with weight loss, which may trigger an unspecific general immunometabolic shift with impact on autoimmunity. However, we neither observed an increase in plasma levels of active ghrelin nor of corticosteroids in DTR mice indicating that the short-term trp-free diet did not induce strong unspecific stress or starvation signals. We did, however, observe a striking drop in plasma leptin levels in DTR mice as commonly observed during nutrient restriction^45^. While administration of leptin aggravates autoimmune neuroinflammation^46^, treatment with leptin-neutralizing antibodies results in amelioration of EAE in mouse models, which is associated with a suppression of encephalitogenic T cells^47^. Hence, we cannot fully exclude that the reduction of plasma leptin may contribute to DTR-mediated attenuation of autoimmune neuroinflammation in our model.

Trp metabolism exerts numerous immunomodulatory functions^48^. Hence, it is tempting to speculate that alterations of metabolite levels by DTR may explain the observed phenotype. Dietary trp is endogenously metabolized in the mammalian intestine through two distinct pathways, the kynurenine pathway (KP) via IDO1 expressed in intestinal immune or epithelial cells and through the serotonin pathways via trp hydroxylase (TpH1) expressed in enterochromaffin cells^49^. Several lines of evidence argue against a causal role of the endogenous intestinal catabolism of dietary trp: (1) Exogenous administration of 5-HTP fails to break resistance of DTR mice to EAE. (2) Genetic ablation of GCN2, the key sensor of trp depletion, fails to break resistance of mice to DTR. (3) Systemic (i.p.) trp reconstitution is not able to overcome the protective effects of DTR.

Instead, our data show that the suppressive effect of DTR on EAE is dependent on the presence of microbiota, as GF mice do not benefit from DTR in the EAE model. We observed a complex change in the gut microbiome induced by DTR, which—in line with other studies^22^—includes an enrichment of trp-synthesizing bacterial genera. This endogenous regulatory shift likely involves the competitive advantage of such bacteria when dietary trp is not present, which in turn may also lead to altered bacterial trp catabolite levels, e.g. via the tryptophanase–indole pathway. These catabolites are known to exert a plethora of immunomodulatory functions in various experimental settings including EAE^19,20^ via the AHR pathway. For instance, trp-derived microbial metabolites protect from metabolic syndrome^50^, experimental colitis^51,52^, and limit CNS autoimmunity by targeting the AHR on astrocytes and microglia^19,20^. In addition, systemic administration of a synthetic trp metabolite demonstrated to activate the AHR^53^ suppresses autoimmune neuroinflammation in mice^54^. However, in our model neither genetic ablation nor supplementation with AHR-activating indoles modulated the suppressive effects of DTR. Of note, as we used a murine model with a constitutive AHR-deletion we cannot exclude that opposing functions of the AHR in different cell populations may have caused this seeming lack of effect of the receptor deletion. Although we used great care to employ biologically active concentrations of the respective AHR ligands, our setup may not fully reflect the spatial distribution of ligands, which is needed for modulation of the DTR effect. Still, our results clearly suggest the presence of an AHR-independent, microbiota-mediated DTR mechanism, which suppresses distant autoimmunity in the CNS.

We obeserved marked colonic inflammation in response to the DTR regimen employed in this study accompanied by a shift in the gut microbial ecology. It is a well-accepted concept that interference with intestinal homeostasis affects systemic immunity and that the number and phenotype of extra-intestinal T cells is shaped by the gut microbiome^1,8–10,55^. Our data suggest that DTR does not affect priming of MOG-reactive T cells, but exerts its impact by both reducing the number of circulating MOG_35–55_-reactive activated CD4^+^ T cells as well as their migratory properties. Transcriptomal profiling of peripheral MOG-reactive T cells also revealed an altered phenotype, with an increase in *Il2ra*, *Il2rb*, *Nptn*, and *Furin* mRNA levels in response to DTR, while *Il10ra* and *Crcp* expression levels were reduced. The induction of IL-2R transcripts in response to DTR is compatible with a distinct hyperproliferative phenotype of MOG-reactive T cells. CD4+ T cells lacking Nptn display enhanced TCR-mediated cytokine production^56^, indicating that Nptn restricts T cell activation. Further studies will have to clarify and deconvolute the exact mechanistic influence of DTR on the complex transcriptional network of encephalitogenic T cell responses.

Our results critically mandate the identification of essential microbial species and trp metabolites that inhibit autoreactive T cell responses, also beyond the CNS. Revealing DTR-induced alterations in T cell-intrinsic signaling pathways that interfere with the formation and migration of encephalitogenic immune cells and validating key findings in patient samples will offer the potential to translate our findings into therapeutic approaches for the treatment of MS patients.

## Methods

### Animals

C57BL/6J wild-type (WT) mice were purchased from Charles River or Janvier Labs. GCN2 knockout (*Gcn2*^*−/−*^; B6.129S6-*Eif2ak4*^*tm1.2Dron*^/J*)* and AHR knockout (*Ahr*^*−/−*^; B6.129-*Ahr*^*tm1Bra*^/J) mice were ordered from Jax® Mice, The Jackson Laboratory and bred at the animal facility of the DKFZ Heidelberg. 2D2 mice, which harbor a transgenic TCR against MOG_35–55_, were originally obtained from Jax® Mice, The Jackson Laboratory (C57BL/6-Tg(Tcra2D2,Tcrb2D2)1Kuch/J) and bred at the DKFZ. C57BL/6J Ly5.1 mice (B6.SJL-*Ptprc*^*a*^ *Pepc*^*b*^/BoyJ) were provided by the Center for Preclinical Research of the DKFZ Heidelberg. 2D2 × Ly5.1 mice were generated in-house by crossing 2D2 mice to C57BL/6J Ly5.1 mice. The presence of the congenic marker Ly5.1 was determined by standard flow cytometry for CD45.1. Mice harboring one transgenic allele of the transgenic TCR were used for subsequent experiments. GF C57BL/6J mice were a kind gift of Christoph Reinhardt (Translational Research of Thrombosis & Hemostasis, Mainz). Sex-, body weight- and age-matched mice were used for further experiments. If not stated otherwise, female mice were used for the experiments. All mice were 6–14 weeks of age at use. Mice were kept under SPF conditions at the animal facility of the DKFZ Heidelberg or in the GF facility in the TARC, University Medical Center Mainz. Experiments were performed according to the rules of the German Animal Welfare Act and were licensed by the regional authority Karlsruhe. Genotyping was performed using the following primers (all purchased from Sigma-Aldrich 5′–3′ forward, reverse): *Gcn2*^*−/−*^ (TCT CCC AGC GGA ATC CGC ACA TCG, ATC CAG GCG TTG TAG TAG CGC ACA), 2D2 (CCC GGG CAA GGC TCA GCC ATG CTC CTG, GCG GCC GCA ATT CCC AGA GAC ATC CCT CC), *Ahr*^*−/−*^ (TGG ATG TGG AAT GTG TGC GAG, GGA TTT GAC TTA ATT CCT TCA GCG GG).

### Active and passive EAE induction

For active EAE induction mice were immunized s.c. at the lateral pectoral regions with 2 × 100 µg murine MOG_35−55_ peptide (MEVGWYRSPFSRVVHLYRNGK; GenScript) emulsified in CFA (BD DifcoTM) containing 4 mg/ml *Mycobacterium tuberculosis* H37 Ra (BD DifcoTM). Two i.p. injections of 200 ng pertussis toxin (List Biological Laboratories) in PBS were given 0 and 2 days post-immunization. The mice were observed for clinical signs and scored using a 14-stage scoring system ranging from 0 to 5 (Supplementary Table 1). In accordance with the German Animal Welfare Act mice with a score of 4 were immediately sacrificed by cervical dislocation or perfusion in deep anesthesia (Ketavet/Rompun). The cumulative score is defined as the total sum of clinical EAE scores for each individual mouse.

For passive EAE induction, donor mice were immunized as described above. Nine to 11 days after immunization splenocytes and lymph node cells were isolated and restimulated in vitro for 3 days in the presence of 10 µg/ml MOG_35–55_. In all, 10 ng/ml IL23 (R&D), 40 mM NaCl, and 10 µg/ml anti-IFNγ (XMG1.2; eBioscience) were added in addition to achieve T_H_17 polarization. CD4^+^ T cells were enriched using standard negative depletion by MACS (Miltenyi Biotec) on an autoMACS Pro Separator (Milteny Biotec) according to the manufacturer’s instructions. To this end, the following biotinylated antibodies were used: anti-CD8a (53–6.7; eBioscience), anti-CD19 (6D5; BioLegend), anti-CD49b (DX5; BioLegend), anti-CD11c (N418; BioLegend), anti-CD11b (M1/70; BioLegend), anti-Ter119 (Ter119; eBioscience). Mice received 2 × 10^6^ cells i.v. and clinical signs were evaluated as described above.

### Feeding experiments

For feeding experiments mice were fed with a custom-made protein-proficient (+protein #12450B) diet that served as a control for a protein-deficient (−protein diet #D12051001) diet synthesized by Research Diets. Trp-free (−trp #12083001) and met-free (−met #A14042201) diets were produced on the basis of the trp- and met-proficient control diet (+trp #A08051501). During feeding experiments all mice were fed with the control diet for 1–2 weeks prior to immunization. Protein- or single amino acid-free diets were applied on the day of immunization until the end of experiment if not indicated otherwise. Weight loss was routinely monitored and did not exceed 30% for more than three consecutive days. In the case of i.p. and p.o. trp supplementation 2.5 mg trp was either injected i.p. in PBS or applied in the drinking water every day. For 5-hydroxytryptophan (5-HTP) rescue experiments mice received daily i.p. injections of 40 mg/kg 5-HTP. Supplementation with the AHR ligand indole-3-aldehyde (I3A; Sigma-Aldrich) or indole-3-carbinole (I3C; Sigma-Aldrich) was performed by daily oral gavage at a dose of 18 mg/kg in 0.5% methylcellulose (Sigma-Aldrich).

### Isolation of lymphocytes from spleen, LN, and CNS

Spleens and lymph nodes were excised and meshed twice through a 70 μm cell strainer to obtain a single-cell suspension and, for splenocytes, erythrocytes were lysed with lysis buffer (150 mM NH_4_Cl, 10 mM KHCO_3_, and 100 μM Na_2_EDTA) for 2 min at room temperature. For isolation of CNS-infiltrating lymphocytes, mice were cardially perfused with PBS in deep anesthesia. Spinal cord tissue was excised, mechanically dissected, dispersed in HBSS (Sigma-Aldrich) supplemented with 50 µg/ml Liberase DL (Roche) and incubated for 45 min at 37 °C under slow rotation. Single-cell suspensions were subsequently meshed through a 100 and 70 µm cell strainer, stained, and analyzed by flow cytometry.

### Analysis of plasma amino acids and corticosteroids levels

The amino acid and corticosterone concentrations in plasma were measured by electrospray ionization tandem mass spectrometry (ESI-MS/MS) using a Quattro Ultima triple quadrupole mass spectrometer (Micromass, Manchester, UK) equipped with an electrospray ion source and a Micromass MassLynx data system. For this purpose 5 μl of the plasma samples were transferred onto a 4.7 mm filter paper punch, dried at RT overnight, and extracted with 100 μl of deuterium-labeled standard solution in methanol. After 20 min, the samples were centrifuged and the extract was evaporated to dryness, reconstituted in 60 µL of 3 N HCl/butanol, placed in sealed microtiter plates, and incubated at 65 °C for 15 min. The resulting mixtures were dried, and each residue was finally reconstituted in 100 µL solvent of acetonitrile/water/formic acid (50/50/0.025, v/v/v) prior measurement.

### D-Trp analysis in fecal and plasma samples by chiral HPLC

Mice feces was mixed at a ratio of 1:2 (w/v) with 80% methanol/water (v/v) at 4 °C. Mice plasma samples were thawed on ice and mixed at a 1:1 ratio (v/v) with 80% methanol/water (v/v). Sample extraction was performed as described above and 30 µl of the obtained supernatants were injected into the HPLC for analysis. A Dionex Ultimate® 3000 uHPLC (Thermo Scientific) was used for chromatographic separation of D-Trp. This was achieved on an Astec® CHIROBIOTIC® T Chiral HPLC column, 25 cm × 4.6 mm (Sigma-Aldrich™) with 5 µm particle size. The mobile phase had a flow rate of 1 ml/min, comprised methanol (30%), water (70%), and formic acid (0.02%). D-Trp was detected based on comparison with standards, of their respective retention times and UV emission spectra at 205 nm. Chromeleon™ 7.2 Chromatography Data System (Thermo Scientific™ Dionex™) was utilized to analyze the obtained results.

### Leptin, active ghrelin, and serotonin ELISA

Commercial ELISA kits for detection of leptin (mouse leptin ELISA kit; Sigma-Aldrich) and active ghrelin (rat/mouse ghrelin (active) ELISA; Sigma-Aldrich) in plasma samples were purchased and performed according to the manufacturer’s instructions. OD was measured at 450 and 595 nm using the iMark^TM^ Microplate Reader (Bio-Rad).

A commercial ELISA kit for detection of serotonin (IDK® Serotonin ELISA) was purchased from Immundiagnostik AG and performed according to the manufacturer’s instructions. OD was measured at 450 and 620 nm using a BMG CLARIOstar plate reader.

### Auto-MOG antibody ELISA

For anti-MOG IgG1 and IgM plasma ELISA, high-binding 96-well plates were coated with 0.25 µg/well MOG_1–125_ (Anaspec) or 0.25 µg/well BSA in 50 µl PBS overnight at 4 °C. On the next day plates were washed with PBS and free binding sites were blocked with ELISA blocking buffer (3% FBS, 0.05% Tween-20 in PBS) for 1 h at RT. Samples were diluted 1:100 in blocking buffer and 50 µl were added to the plates. Plates were incubated at 4 °C overnight. Secondary anti-mouse IgG1-HRP (Bethyl; A90-205P) and anti-mouse IgM (Sigma-Aldrich; A8786–1ML) were added at a 1:5000 dilution in ELISA blocking buffer and plates were incubated at RT for 1 h. After five washes 50 µl/well TMB solution (eBioscience) were added. The reaction was stopped with 25 µl 1 M H_2_SO_4_ after 3–10 min and the OD was measured at 450 and 595 nm. OD values were corrected by substraction of a control protein (BSA).

### Trp analysis in fecal and plasma samples by UPLC-ESI-HRMS/MS

Mice feces was thawed on ice, transferred into microcentrifuge tubes, and the weight of the samples were taken (wet weight) to adjust the sample/extraction solvent mix ratio of 1:5 (w/v) using 80% methanol/water (v/v) at 4 °C. Mice plasma samples were thawed on ice and mixed with 200 µl 80% methanol/water (v/v) at 4 °C. Samples were extracted by a 120 min incubation period by shaking at 4 °C. Next, sample extractions were centrifuged for 20 min at 4 °C using an Eppendorf benchtop centrifuge at maximum speed. The cleared supernatants were collected after centrifugation at RT for 20 min and 5 µl were used for ultrahigh-performance liquid chromatography-electrospray ionization-high-resolution mass spectrometry/mass spectrometry (UPLC-ESI-HRMS/MS).

UPLC-ESI-HRMS/MS analysis was performed with an UltiMate 3000 system (Thermo Fisher) with a Waters ACQUITY UPLC BEH C18 Column, 130 Å, 1.7 µm, 2.1 mm × 50 mm coupled to an Impact II qTof mass spectrometer (Bruker) with MeCN/0.1% formic acid in H_2_O (5:95% over 5 min followed by 5:95 → 95:5% over 9 min followed by 95:5% over 2 min, flow rate 0.4 ml/min). A 10 mM sodium formate solution served as an internal mass calibrant. The following MS settings were used: (i) source settings: capillary voltage 4500 V, nebulizer gas pressure (nitrogen) 3 bar, ion source temperature 200  °C, dry gas flow of 8 l/min; (ii) general scan settings: ion polarity positive, mass range 100–1200 *m/z*, spectra rate 3 Hz (MS and MS/MS); (iii) tune parameters: transfer funnel 1 RF 300 Vpp, Funnel 2 RF 300 Vpp, isCID off, hexapole RF 60 Vpp, stepping settings 20–50 keV; (iv) MS/MS settings: eight precursor ions, threshold 1000 cts. (absolute), activated active exclusion after three spectra and 0.5 min release time, active precursor reconsidering factor 4, smart exclusion two times.

A trp standard stock solution (2 mg/ml in H_2_O) was used to generate dilutions in a range from 1:100 to 1:1.000. Five microliters of these samples were measured in technical triplicates using UPLC-ESI-HRMS/MS analysis. Signal area counts of EIC *m/z* 205.097 [M + H]^+^ were plotted against concentration resulting in a linear function (*R*^2^ = 0.99) used for quantification.

### Histology, immunohistochemistry, and Evan’s Blue assay

Dissected spinal cords were fixed in 4% PFA in PBS overnight at 4 °C and embedded in paraffin. After deparaffination and rehydration, antigen-retrieval was performed by boiling 10 µm sections in 10 mM citric acid buffer, pH 6.0. Endogenous peroxidase activity was blocked with 3% H_2_O_2_ in PBS. Sections were covered with blocking solution (10% goat plasma, 2% BSA in PBS), followed by incubation with different primary antibodies overnight at 4 °C: anti-CD3 (polyclonal; Dako) and anti-CD107b (M3/84; BioLegend). The secondary antibody (goat anti-rat/rabbit IgG; Vector Laboratories) was applied to the sections in blocking buffer for 1 h at RT. HRP-coupled streptavidin (VECTASTAIN® Elite ABC kit; Vector Laboratories) was used to detect the biotinylated secondary antibody. The sections were then exposed to the ultraView™ Universal DAB Detection Kit (Dako) according to the manufacturer’s protocol and counterstained with hematoxylin (Roth). Quantitative analysis of T lymphocyte (CD3) or macrophage (CD107b) infiltration was quantified using Fiji software. Several sections per animal were analyzed and the mean percentage of infiltrated spinal cord area was calculated for the different groups.

For detection of myelin degradation standard Luxol Fast Blue (LFB) staining was performed and demyelination was quantified as described previously^57^. All images were acquired using a Zeiss Cell Observer.Z1 widefield microscope in combination with a ×20/0.3 Plan-NEO Ph1 DICIII objective. ZEN software was used to control image acquisition.

In order to assess BBB disruption, mice were injected i.v. with 200 µl 0.5% Evan’s Blue (Sigma-Aldrich) 2 h before the spinal cord was excised. Tissue was incubated in formamide for 24 h at 55 °C. Afterwards, samples were centrifuged at 10,000 × *g* to pellet debris. The absorbance of the supernatant and standard dilutions of Evan’s Blue was measured at 595 nm using an iMark^TM^ Microplate Reader (Bio-Rad) and the Microplate Manager 6 software.

### Ex vivo T cell proliferation and cytokine secretion assay

For the analysis of antigen-specific proliferation of T cells, mice were sacrificed on indicated days after immunization. If applicable, splenocyte single-cell suspensions were stained with the CellTrace™ Far Red Cell Proliferation Kit or CFSE Cell Proliferation Kit (Invitrogen) according to the manufacturer’s instructions. Cells were restimulated with 1, 10, or 20 µg/ml MOG_35–55_. As an irrelevant peptide control cells were stimulated with 10 or 20 μg/ml isocitrate dehydrogenase 1 **(**IDH1_123–142_; GWVKPIIIGRHAYGDQYRAT, Research Group GMP & T Cell Therapy, DKFZ Heidelberg; custom-made); 2 µg/ml Concanavalin A (ConA; Sigma-Aldrich) was used as a positive control. After 72 h cells were harvested and stained for flow cytometry using a live/dead cell discrimination dye and antibodies against CD3, CD4, and CD8a. CellTrace Far Red dye dilution was measured to assess proliferation rates. All cells with at least one division were counted as proliferating cells. Supernatants were collected to measure IL17A by standard cytokine ELISA using the mouse IL17A ELISA Ready-SET-Go!^®^ Kit (eBioscience) according to the manufacturer’s instructions.

### In vivo T cell proliferation assay

CD4^+^ T cells were purified from splenocytes and lymph node cells from naïve 2D2 mice by magnetic separation. In brief, single-cell suspension were labeled with biotinylated antibodies targeting CD8a, CD19, CD49, CD11c, CD11b, and Ter119. Standard negative depletion was performed using MagniSort™ Streptavidin Negative Selection beads (Invitrogen) as decribed in the technical data sheet provided by the company. Purified CD4^+^ T cells were stained with the CellTrace™ Far Red Cell Proliferation kit (Invitrogen) according to the manufacturer’s instructions. 5 × 10^6^ cells were injected i.v. into naïve C57BL/6J Ly5.1 mice, followed by active EAE induction 18 h after transfer. Six days after transfer blood, draining lymph nodes and spleens were excised and dye dilution in CD45.2^+^ CD4^+^ T cells was measured using flow cytometry to assess in vivo proliferation.

### Flow cytometry and intracellular cytokine staining (ICS)

For detection of cytokine secretion, cell suspensions were incubated with MOG_35–55_, PLP_139–151_, or IDH1_123–142_ peptide for 2 h prior to addition of 5 µg/ml Brefeldin A (Sigma-Aldrich) or 1× Monensin Solution (BioLegend) for GM-CSF and IL10.

Single-cell suspensions were stained with the following fluorescent antibodies to surface antigens: PE-Cy7 anti-mouse CCR6 (29-2L17; BioLegend), APC anti-mouse CD3 (17A2; BioLegend), Brilliant Violet 711 anti-mouse CD3 (17A2; BioLegend), eFluor^TM^ 450 anti-mouse CD3 (17A2; eBioscience), FITC anti-mouse CD3 (17A2; BioLegend); APC anti-mouse CD4 (RM4-5; BioLegend), eFluor^TM^ 506 anti-mouse CD4 (RM4-5; eBioscience), FITC anti-mouse CD4 (GK1.5; eBioscience), Pacific Blue anti-mouse CD4 (RM4-5; BioLegend), PerCP-Cy5.5 anto-mouse CD8a (53-6.7; eBioscience), Brilliant Violet 421 anti-mouse CD44 (IM7; BioLegend), Brilliant Violet 510 anti-mouse CD45 (30-F11; BioLegend), PE-Cy7 anti-mouse CD45.1 (A20; BioLegend), PE anti-mouse CD45.2 (104; BioLegend), FITC anti-mouse CXCR3 (CXCR3-173; eBioscience) and Streptavidin FITC (BioLegend). Dead cells were excluded using the Fixable Viability Dye eFluor^TM^ 780 (eBioscience). For tetramer stainings single-cell suspension were stained with T-Select I-Ab MOG 35–55 Tetramer-PE (MBL) or T-Select I-Ab OVA 323–339 Tetramer-PE (MBL) as a negative control tetramer at a 1:10 ratio in PBS supplemented with 50% FCS and 2 mM EDTA for 30 min at room temperature prior to surface antigen staining.

For intracellular staining, cells were stained using the Intracellular Fixation & Permeabilization Buffer Set (eBioscience) or the Foxp3/Transcription Factor Staining Buffer Set (eBioscience) according to the manufacturer’s instructions and the following fluorescently labeled antibodies against intracellular molecules: PerCP-eFluor^TM^ 710 anti-mouse CD40L (MR1; eBioscience), FITC anti-mouse FoxP3 (FJK-16s; eBioscience), PerCP-Cy5.5 anti-mouse GM-CSF (MP1-22E9; BioLegend), FITC anti-mouse IFNγ (XMG1.2; eBioscience), PE anti-mouse IFNγ (XMG1.2; eBioscience), PE anti-mouse IL-10 (JES5-16E3; eBioscience), PE-Cy7 anti-mouse IL17A (eBio17B7; eBioscience).

For quantification of immune cell counts, 123 count eBeads (eBioscience) were added prior to sample acquisition. In the case of transmigration assays Flow-Count Fluorospheres (Beckman Coulter) were used.

Flow cytometry acquisition was performed on FACSCanto II (BD Biosciences), Attune NxT (Thermo Fisher), or Gallios (Beckman Coulter) for transmigration assays. Data were analyzed using FlowJo X or Kaluza software 2.1 (Beckman Coulter) for transmigration assays. Frequency of cytokine-secreting and CD40L-expressing cells was corrected by substraction of a control peptide (PLP_139–151_ or IDH1_123–142_).

### Transmigration assay

T cells were purified from splenocytes from naïve C57BL/6J mice by magnetic separation using the Pan T Cell Isolation Kit II, mouse kit (Miltenyi) according to the manufacturer’s instructions. T cells were stimulated with 2 µg/ml anti-CD3 (145-2C11; BioLegend) and 1 µg/ml anti-CD28 (37.51; BioLegend) and cultured for 2 days in standard T cell medium supplemented with plasma from naïve C57BL/6J mice or EAE animals at a 2:1 ratio.

MBMECs were isolated from 10 8–12-week-old female C57BL/6 mice. In brief, meninges were removed from cortex and tissue was mechanically homogenized and digested with 1 mg/ml collagenase type II (Worthington) and 20 µg/ml DNase I (Sigma-Aldrich) for 1 h at 37 °C. Myelin was removed by centrifugation in DMEM supplemented with 20% BSA for 20 min at 1000 × *g*, 4 °C, followed by a second digestion step in 1 mg/ml Collagenase/Dispase (Roche) and 10 µg/ml DNase I for 1 h at 37 °C. After ultracentrifugation on a Percoll gradient (Sigma-Aldrich; 16000 r.p.m., 1 h, 4 °C), fragments of capillaries were seeded onto 24 wells pre-coated with 40% Collagen from human placenta type IV (Sigma-Aldrich) and 10% fibronectin (Sigma-Aldrich). MBMECs were cultured at 37 °C, 5% CO_2_ and after 4 days fresh, puromycin-free medium was added. Two days later, when MBMEC layers reached confluence cells were used for subsequent experiments.

Six days after isolation, MBMECs were trypsinized, resuspended, and seeded at a density of 2 ×10^4^ cells per pre-coated transwell inserts (pore size 3.0 µM; Corning). Transendothelial electrical resistance (TEER) measurements were performed using the cellZscope 24-cell module and cellZscope v2.2.2 Software (nanoAnalytics GmbH). Measurements were taken for 4 to 5 days until the MBMEC monolayer reached full confluence (Ccl at <1 µF/cm^2^ and TEER at its maximum plateau level). At that point, cells were inflamed with each 50 U/ml TNFα (Peprotech) and IFNγ (Peprotech) for 24 h. Afterwards MBMECs were washed and pre-stimulated T cells resuspended in RPMI were added to the apical side of the transwell insert. Migration medium (80% RPMI, 20% FCS, 2% B27 (Gibco)) was added to the lower chamber and T cells were left to migrate overnight at 37 °C, 5% CO_2_. Migrated T cells were harvested from the lower chamber and analyzed by flow cytometry.

### Gene expression analysis of MOG_35–55_-reactive CD4^+^ T cells

For adoptive transfer of MOG_35–55_-reactive CD4^+^ T cells single-cell suspension of splenocytes and lymph node cells from naïve 2D2 × Ly5.1 mice were subjected to magnetic separation. In a first step CD4^+^ T cells were enriched using the CD4+ T Cell Isolation Kit, mouse (Miltenyi Biotec) according to the manufacturer’s protocol with additional supplementation of 5 µl biotinylated anti-CD8 (53–6.7; eBioscience) per 10^7^ cells, followed by positive selection of naïve CD4^+^ T cells with CD62L Microbeads, mouse (Miltenyi Biotec) as described in the technical data sheet provided by the company. 2 × 10^6^ naïve CD4^+^ T cells were injected i.v. into recipient mice. Eighteen hours after cell transfer mice were immunized for EAE induction as described above. One group received a trp-free diet from the day of immunization on.

Seven days post EAE-induction spleens were excised after cervical dislocation and single-cell suspensions were enriched for T cells using the MagniSort Mouse T Cell Enrichment Kit (eBioscience) according to the manufacturer’s protocol, followed by a surface stain against CD3 (APC, 17A2; BioLegend), CD4 (Pacific Blue, RM4-5; BioLegend), and CD45.1 (PE-Cy7, A20; BioLegend). Dead cells and contaminating lineage-negative, biotin-positive cells were removed by staining with Fixable Viability Dye eFluor^TM^ 780 (eBioscience) and Streptavidin FITC. Viability Dye^−^ FITC^−^ CD3^+^ CD4^+^ CD45.1^+^ cells were sorted directly into 1× Extraction Buffer (PicoPure™ RNA Isolation Kit; Applied Biosystems^TM^) in low-binding tubes using a BD Aria II instrument. RNA extraction was performed immediately after cell collection according to the manufacturer’s protocol including DNase I treatment.

Gene expression analysis was performed using the nCounter Autoimmune Profiling Panel with the nCounter NanoString™ technology (NanoString Technologies, Seattle, WA). The nCounter technology allows for multiplexed gene expression analysis based on simultaneous hybridization and digital quantification of fluorescently labeled oligonucleotide probes, so called CodeSets^58^. All RNA samples were quantified by using Qubit^TM^ and quality control was performed using the Agilent 2100 Bioanalyzer system. Qualified samples were subjected to nCounter analysis as recommended by the manufacturer. In brief, 25 ng of total RNA were used as input material for the CodeSet hybridization at 65 °C overnight. Readout of the experiment was performed using the SPRINT^TM^ Profiler from NanoString Technologies (Seattle, WA). The quality control of the NanoString data was performed using nSolver Analysis Software 4.0. The data were normalized against the housekeeping genes using the R package NanoStringNorm^59^. The genes that were undetectable (0 after normalization) in more than half of the samples of each group (control and trp-free) were excluded from the analysis. This resulted in the removal of 250 genes from the dataset. The remaining genes were tested for differential expression using the Bioconductor package NanoStringDiff^60^. Genes with FDR adjusted *p* value of 0.05 were considered to be differentially expressed.

### Microbiota analysis

Total genomic DNA was extracted from ileal and colonic fecal pellets using the DNeasy PowerSoil Kit (Qiagen). Extracted DNA was used to amplify the 16S rRNA gene variable region V3–V4. Amplification performance was ascertained by running an aliquot on a 2% agarose gel. Amplicon quantities were normalized using the SequalPrep™ Normalization Plate Kit (Invitrogen), pooled to make a library and sequenced using the MiSeq Reagent Kit v3 (Illumina) at Institute of Clinical Molecular Biology, Kiel, Germany

Analysis of sequencing data was conducted using an in-house shell script pipeline based on standard procedures for 16S rRNA gene sequence data^61^. In brief, the multiplex identifiers (MID) and primer sequences were removed prior to the sequence trimming and refined quality-controlled reads were aligned against mothur curated SILVA reference database. Reads not aligned against 16S rRNA gene V3–V4 region were removed from subsequent analysis. Finally, a distance matrix was computed using these aligned reads, and binned into species level (97%) operational taxonomic units (OTU). Phylogenetic assignment of species OTUs as well as at higher taxonomical level genus and phyla was accomplished by mapping the sequences against mothur curated RDP database (version 16). Further analyses including α- and β-diversity estimates was performed with mothur. Non-parametric permutational multivariate analysis of variance (NPMANOVA) was performed for ascertaining the significance of clustering in sampling groups for β-diversity^62^.

In order to infer the functional potential of microbial communities we employed PICRUSt^37^. In brief, 16S rRNA gene sequences were mapped against mothur curated GreenGenes reference database (gg_13_5_99). Subsequently, the normalized shared phylotype file was used to generate a biom file in mothur. The shared biom file was uploaded to the PICRUSt galaxy terminal (http://huttenhower.sph.harvard.edu/galaxy/) to infer the functional metagenomics potential of bacterial communities. Differences in the predicted metagenomic potential were tested using non-parametric Mann–Whitney *U*-test with Benjamini–Hochberg correction.

### RNA isolation, RNA sequencing, and analysis

Total RNA was extracted from frozen colon whole tissues (1 cm of most distal colon) of the mice using the RNeasy Mini kit from Qiagen using standard protocol. The frozen tissue samples were homogenized using Tissue Lyser II for the RNA extraction.

Individual strand-specific single-end sequencing libraries were constructed from the total RNA isolated from the samples using TruSeq Stranded mRNA kit (Illumina Inc.). Sequencing was performed on the Illumina HiSeq3000 (1 × 50 bp) using standard protocols.

Adapters and low-quality bases from the RNA-seq reads were removed using Trim Galore (version 0.4.4)^63^, which is a wrapper tool for Cutadapt^64^ and FastQC^65^. Reads that were shorter than 35 bp after trimming were discarded. The filtered reads were mapped to the mouse genome (GRCm38) using STAR aligner (version 2.5.2b)^66^. featureCounts (version 1.5.2)^67^ was used to estimate the expression counts of the genes. Gene-centric differential expression analysis was performed using the Bioconductor package DESeq2 (version 1.20.0)^68^. Genes with FDR adjusted *p* value of 0.05 were considered to be differentially expressed. DEGs were ranked based on the basemean value to select the top 20 genes for representation in the heatmap format.

Gene ontology enrichment analysis of all significantly DEGs was done using the Bioconductor package topGO (version 2.32.0)^69^ In the topGO analysis, the Fisher.elim *p* value, calculated using the weight algorithm, of 0.05 was used as the significance threshold. Transcription factor-binding site (TFBS) analysis was performed using innateDB (version 5.4)^70^. Principal Component Analysis (PCA) was done using the “prcomp” function implemented in R (version 3.5).

In order to estimate the relative compositions of the immune cells in the mouse colon tissue, cell deconvolution analysis was performed using the ImmuCC webserver^71^. ImmuCC uses the transcriptomal data from the tissue of interest to estimate the relative cell compositions. Raw RNA-seq read counts from all the samples, after filtering for genes that had one or less read in all samples combines, were provided as the input for the ImmuCC analysis. Support Vector Regression (SVR) was chosen as the machine learning method for the analysis.

### Graphical presentation and statistics

All data are presented as means (±SEM for biological replicates, ±SD for technical replicates). Depending on the type of data, significance was assessed with the unpaired two-tailed Mann–Whitney *U* test, the unpaired two-tailed Student’s *t*-test, the paired two-tailed Student’s *t*-test, or the Kruskal–Wallis test. For statistics of sequencing data please refer to the respective section. *p* ≤ 0.05 was considered significant (*p ≤ 0.05, **p ≤ 0.01, ***p ≤ 0.001).

### Reporting summary

Further information on research design is available in the Nature Research Reporting Summary linked to this article.

## Supplementary information

Supplementary Information

Supplementary Dataset 1

Supplementary Dataset 2

Supplementary Dataset 3

Supplementary Dataset 4

Supplementary Dataset 5

Supplementary Dataset 6

Supplementary Dataset 7

Supplementary Dataset 8

Supplementary Dataset 9

Reporting Summary

Source Data

## Data Availability

RNA-seq data that support the findings of this study has been deposited in the GEO repository (GSE127190) and will be made available prior to publication. 16S rRNA gene sequencing data project has been deposited in the European Nucleotide Archive (PRJEB34409). All additional data sets generated or analyzed during this study are included in this published article (and its supplementary information files). The source data underlying Figs. 1b–d, 2a–g, 3a–k, 4a–j, 3a, 3e and Supplementary Figs. 1a–c, 1e–j, 2a–c, 3c, e–f, 4a–c, h–j, 5a–j, 6a–c, e–f are provided as a Source Data file.

## Code availability

The full codes of all scripts are available on reasonable request. For further information on software package versions please refer to the respective Methods section.
