# Supplementary material for: Dietary tryptophan links encephalogenicity of autoreactive T cells with gut microbial ecology
Source: Nat Commun. 2019 Oct 25;10:4877. doi: 10.1038/s41467-019-12776-4 (PMC6814758; doi:10.1038/s41467-019-12776-4)
Supplement: Supplementary file 1 — Supplementary Information [file 41467_2019_12776_MOESM1_ESM.pdf]

Supplementary Information

Dietary tryptophan links encephalogenicity of autoreactive T cells with gut microbial ecology

Sonner et al.

Supplementary Figures

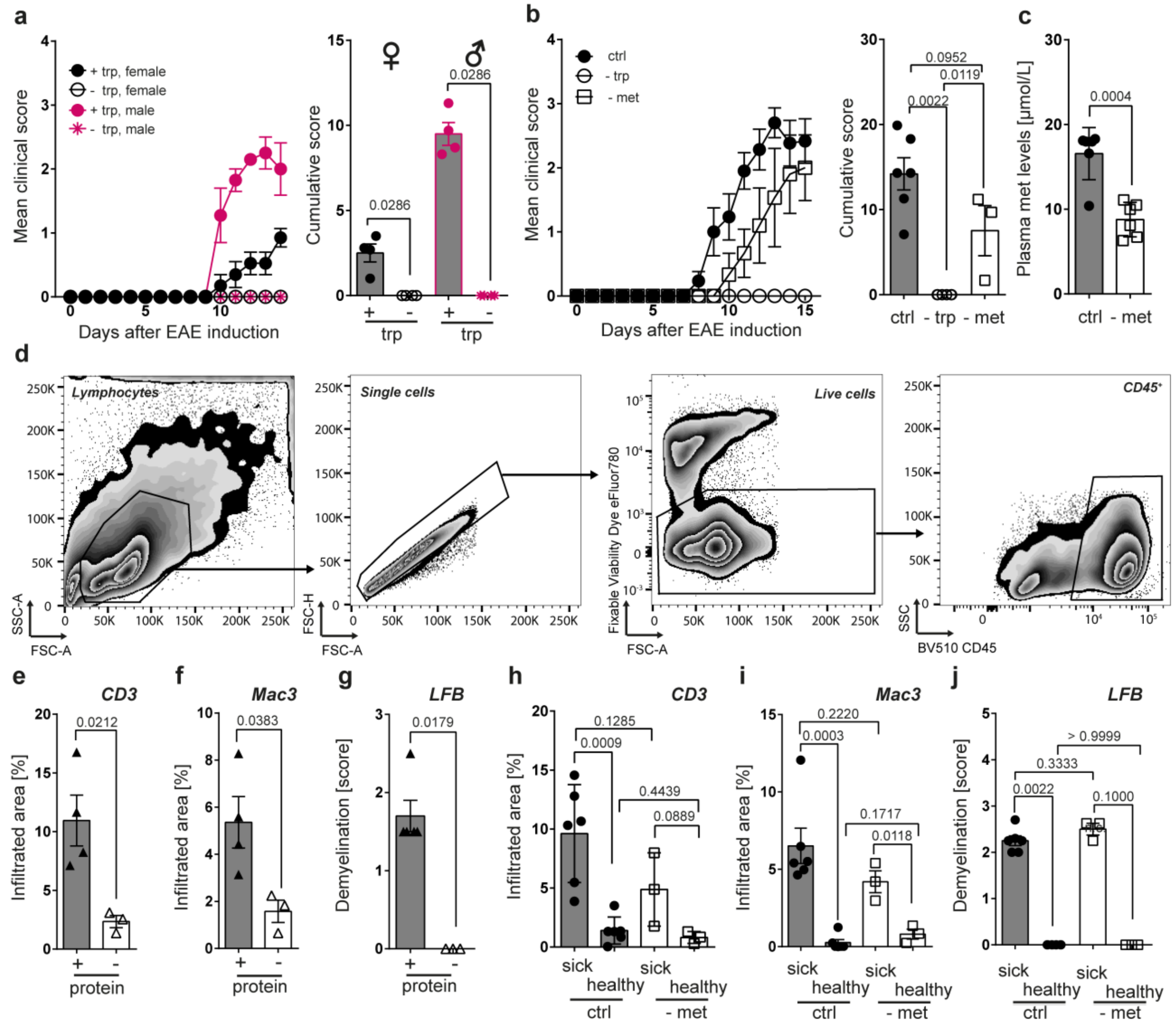

Supplementary Fig. 1 Methionine-free diet partially inhibits EAE. **a** Mean clinical EAE scores and cumulative scores of male and female mice (n = 4). **b** Mean clinical EAE scores and cumulative scores (ctrl: n = 6; -trp: n = 6; -met: n = 3). **c** Plasma met levels of EAE mice (ctrl: n = 6; -met: n = 6). **d** Flow cytometry gating strategy for identification of leukocytes ($CD45^+$) in spinal cord from EAE mice as displayed in **Fig. 2d**. **e-g** Quantitative analysis of **e** T cell and **f** macrophages infiltration, as well as **g** demyelination on spinal cord sections (+protein: n = 4; -protein: n = 3). **h-j** Quantitative analysis of **h** T cell and **i** macrophages infiltration, as well as **j** demyelination on spinal cord sections derived from sick mice, healthy mice (resistant to EAE induction) and both sick and healthy mice that were deprived of met. Statistics: Mann-Whitney *U*-test for **a**, **b**, **g**, **j**; unpaired two-tailed Student's *t*-test for **e**, **f**, **h**, i. Each dot represents one individual mouse. Data are presented as mean ± SEM. Source data are provided as a Source Data file

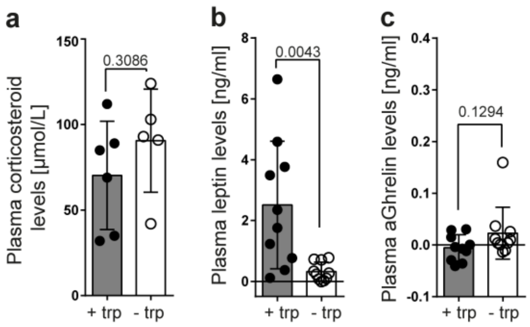

Supplementary Fig. 2 DTR reduces systemic leptin levels. **a** Plasma corticosteroid levels of EAE mice (+trp: *n* = 6; -trp: *n* = 5). **b-c** Plasma levels of **b** leptin and **c** active ghrelin in EAE mice on d14 post-immunization (+trp: n = 10; -trp: n = 10). Statistics: unpaired two-tailed Atudent's *t*-test for **a-c**. Each dot represents one individual mouse. Data are presented as mean ± SEM. Source data are provided as a Source Data file

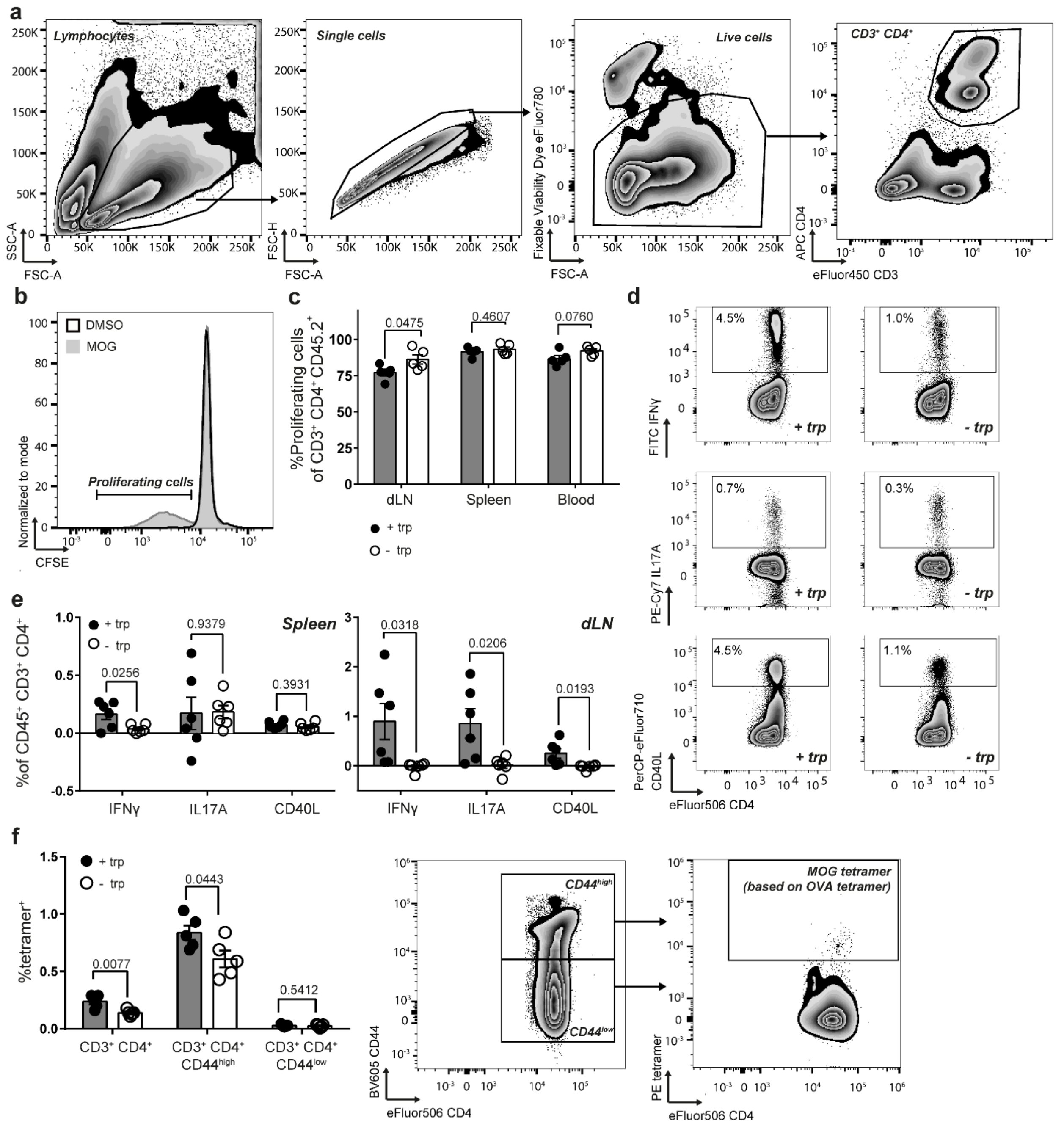

Supplementary Fig. 3 DTR decreases frequency of MOG-reactive T cells in induction phase. **a** Flow cytometry gating strategy for identification of $CD4^+$ T cells as used in **Fig. 3d**, **Fig. 3e**, **Fig. 3g**, **Fig. 3h**, **Supplementary Fig. 3b-f**, **Supplementary Fig. 4a** and **Supplementary Fig. 4c** prior to sub-gating. **b** Gating strategy for identification of proliferating $CD4^+$ T cells. A similar gating strategy was using in **c**. **c** *In vivo* proliferation of 2D2 CD4+ T cells in naïve C57BL/6J recipient mice that received either a trp-free (n = 5) or control (n = 5) diet. Five days post-immunization dLN, spleens and blood were collected to determine the frequency of proliferating $MOG_{35\text{-}55}$-reactive $CD4^+$ T cells within $CD45.2^+$ $CD3^+$ $CD4^+$ cells by flow cytometry. A similar gating strategy was used in **Fig. 3e**. **d** Representative flow cytometry plots for IFNγ and IL17A secretion, as well as CD40L expression on $CD3^+$ $CD4^+$ T cells from **Fig. 3h**. **e** Secondary lymphoid organs were isolated from EAE mice on d7 (n = 6). After *in vitro* restimulation with $MOG_{35\text{-}55}$ or $IDH1_{123\text{-}142}$ expression of INFγ, IL17A and CD40L was measured by intracellular cytokine staining (ICS). **f** Identification of $MOG_{35\text{-}55}$-reactive T cells by tetramer staining of d7 EAE animals (+trp: n = 5; -trp: n = 5). Statistics: unpaired two-tailed Student's t-test for **c**, **e-f**. Each dot represents one individual mouse. Data are presented as mean ± SEM. Source data are provided as a Source Data file

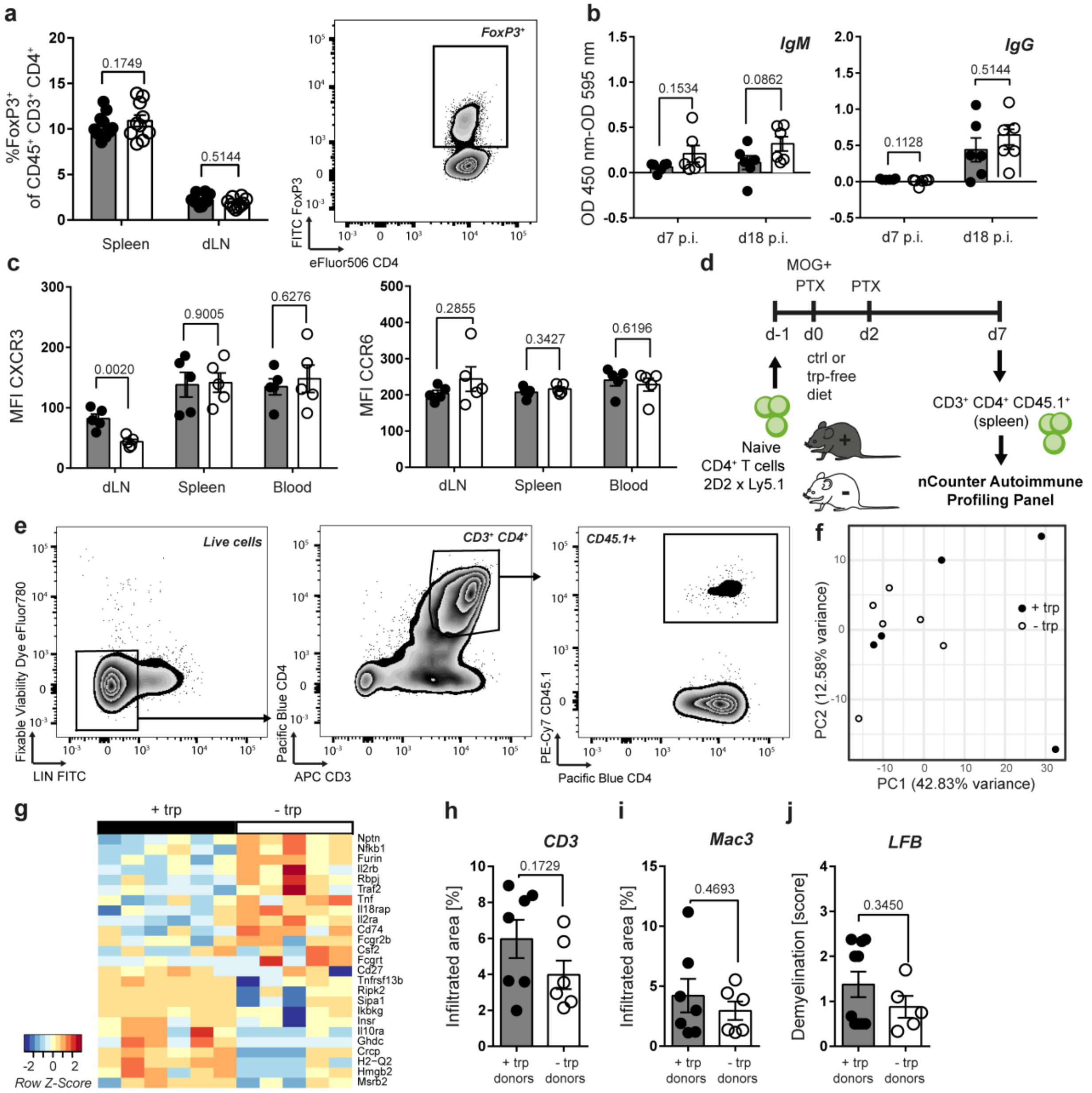

Supplementary Fig. 4 DTR impairs pro-inflammatory T cell responses without T_{reg} induction and antibody involvement. **a** Frequency of FoxP3⁺ CD4⁺ T cells within spleen and dLN in d14 EAE animals (+trp: *n* = 10; -trp: *n* = 10). **b** ELISA for anti-MOG antibody titers at d7 and d18 post-immunization (p.i.) for IgM and IgG in plasma from EAE mice (+trp: *n* = 6; -trp: *n* = 6). **c** Expression of CXCR3 and CCR6 on donor T cells from **Supplementary Fig. 3c** by flow cytometry. **d-g** NanoString gene expression profiling of MOG-reactive 2D2 T cells. $CD3^+$ $CD4^+$ $CD45.1^+$ T cells were subjected to gene expression analysis using the nCounter Autoimmune Profiling Panel. **d** Experimental setup, **e** sorting strategy after exclusion of doublets as illustrated in **Supplementary Fig. 3a**, **f** PCA and **g** heatmap of differentially regulated genes. **h-j** Recipient mice have been transplanted with T cells of immunized donor mice on either a trp-proficient or –deficient diet. Spinal cords of recipients were isolated and stained for **h** T cells (*n* = 7 vs. *n* = 6), **i** macrophages (*n* = 7 vs. *n* = 6) and **j** demyelination (*n* = 9 vs. *n* = 5). Statistics: unpaired two-tailed Student's *t*-test for **a-c**, h, **i**; Mann-Whitney *U*-test for **j**. Each dot represents one individual mouse. Data are presented as mean ± SEM. Source data are provided as a Source Data file or in Supplementary Data 1

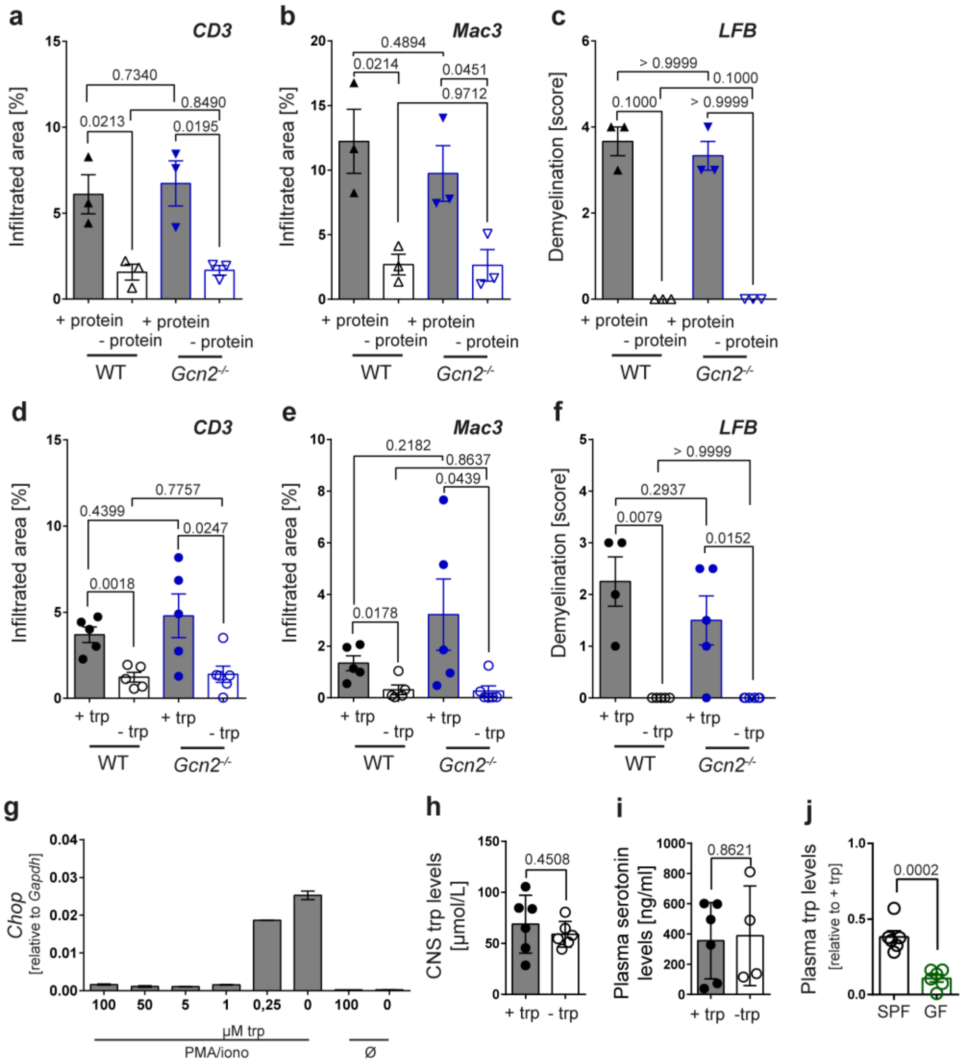

Supplementary Fig. 5 *Gcn2*-deficient mice show similar pathological hallmarks of EAE after DPR or DTR. **a-c** Histological stainings of spinal cords of mice from **Fig. 4a** for **a** T cell infiltration, **b** macrophage infiltration and **c** demyelination. **d-f** Histological stainings of spinal cords of mice from **Fig. 4b** for **d** T cell infiltration, **e** macrophage infiltration and **f** demyelination. Each dot represents one mouse. **g** *Chop* expression after activation of WT T cells. Increasing concentrations of trp were added as indicated. **h** Trp levels in the spinal cord tissue of EAE mice (+trp: n = 6; +-trp: n = 6). **i** Serotonin plasma levels of EAE mice on d14 post-immunization (+trp: n = 6; -trp: n = 4). **j** Plasma trp levels of GF (n = 6) and SPF (n = 6) EAE mice 16 days post-immunization. Ratios between animals that received a trp-free diet and control animals were calculated to estimate the relative drop in trp plasma levels. Statistics: unpaired two-tailed Student's t-test for **a**, **b**, **d**, **e**, **h-j**; Mann-Whitney U-test for **c** and **f**. Each dot represents one individual mouse. Data are presented as mean ± SEM. Source data are provided as a Source Data file

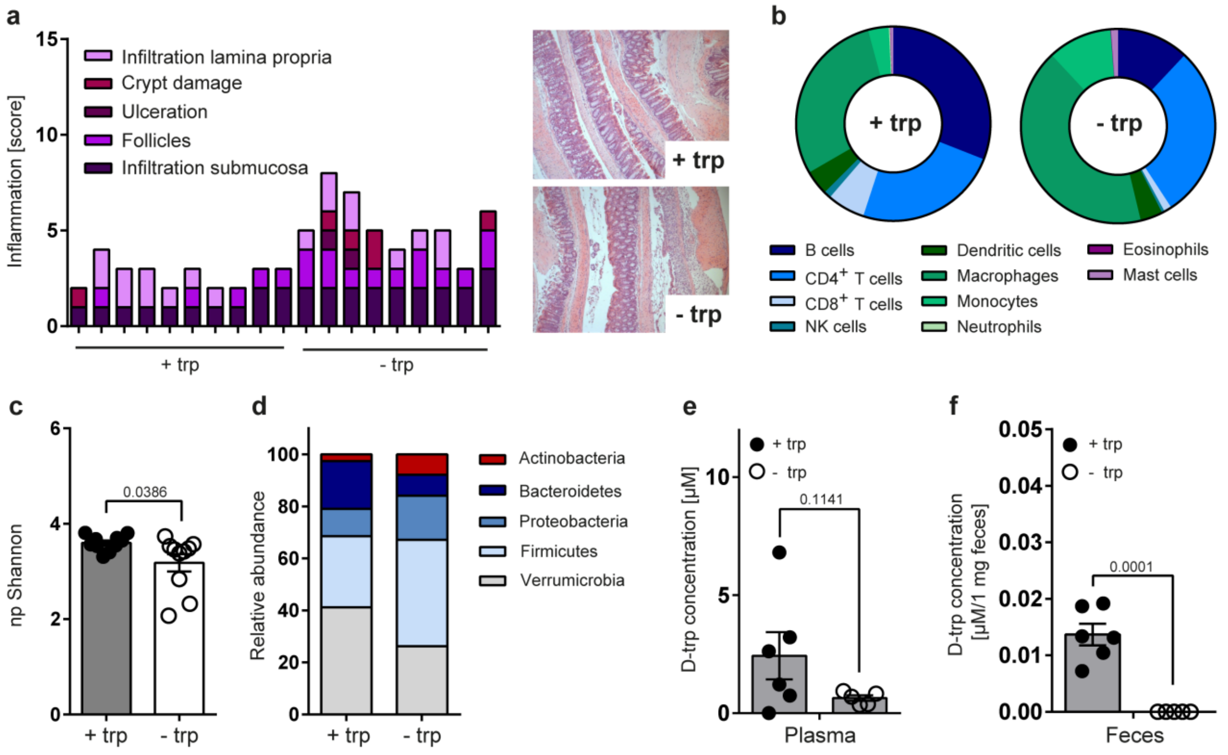

Supplementary Fig. 6 DTR drives Intestinal dysbiosis. **a** Histopathological inflammation in the colon for individual mice from **Fig. 5**. **b** ImmuCC analysis for RNA sequencing samples of colon tissue for mice from **Fig. 5**. **c** Alpha diversity index presented by non parametric (np) Shannon diversity. **d** Relative abundance of major bacterial phyla. **e-f** D-tryptophan concentrations in plasma and feces from EAE mice (+trp: n = 6; -trp: n = 5). **e** Plasma was obtained on d18 post-immunization. **f** Feces was obtained on d9 post-immunization. Statistics: unpaired two-tailed Student's t test in **a**, **c**, **e**, **f**. Each dot represents one individual mouse. Data are presented as mean ± SEM. Source data are provided as a Source Data file or in Supplementary Data 2

Supplementary Tables

Supplementary Table 1: Clinical scoring system for chronic EAE

**Score**	**Clinical signs**
0	No clinical signs
0.7	Partial tail paralysis
1.0	Complete tail paralysis
1.3	Tail paralysis and insecure walking
1.7	Hind limbs only rarely slip through grid
2.0	Hind limbs frequently slip through grid, but secure footsteps still possible<br>No abnormalities inside cage
2.3	Normal walking on grid not possible<br>Evident hind limb paralysis on grid, but hind limb movement clearly visible inside cage
2.7	Paralysis of one hind limb inside the cage
3.0	Complete paralysis of hind limbs, mice drag hind limbs behind symmetrically
3.3	Hip rotation and worsening general impression (pale fur, hunch)
3.7	Front limbs lose their grip to grid when mice are held above cage by their tail<br>Front limbs may slip through grid
4.0	Movement of front limbs inside cage<br>Locomotion inside cage restricted
4.3	Paralysis of one front limb<br>Locomotion inside cage not possible
5.0	Death
