# Supplementary material for: Dietary tryptophan links encephalogenicity of autoreactive T cells with gut microbial ecology
Source: Nat Commun. 2019 Oct 25;10:4877. doi: 10.1038/s41467-019-12776-4 (PMC6814758; doi:10.1038/s41467-019-12776-4)
Supplement: Supplementary file 11 — Reporting Summary [file 41467_2019_12776_MOESM11_ESM.pdf]

nature research

Corresponding author(s): Michael Platten

Last updated by author(s): Sep 19, 2019

Reporting Summary

Nature Research wishes to improve the reproducibility of the work that we publish. This form provides structure for consistency and transparency in reporting. For further information on Nature Research policies, see Authors & Referees and the Editorial Policy Checklist.

Statistics

For all statistical analyses, confirm that the following items are present in the figure legend, table legend, main text, or Methods section.

n/a	Confirmed	
☐	☒	The exact sample size (n) for each experimental group/condition, given as a discrete number and unit of measurement
☐	☒	A statement on whether measurements were taken from distinct samples or whether the same sample was measured repeatedly
☐	☒	The statistical test(s) used AND whether they are one- or two-sided<br>*Only common tests should be described solely by name; describe more complex techniques in the Methods section.*
☒	☐	A description of all covariates tested
☐	☒	A description of any assumptions or corrections, such as tests of normality and adjustment for multiple comparisons
☐	☒	A full description of the statistical parameters including central tendency (e.g. means) or other basic estimates (e.g. regression coefficient) AND variation (e.g. standard deviation) or associated estimates of uncertainty (e.g. confidence intervals)
☒	☐	For null hypothesis testing, the test statistic (e.g. F, t, r) with confidence intervals, effect sizes, degrees of freedom and P value noted<br>*Give P values as exact values whenever suitable.*
☒	☐	For Bayesian analysis, information on the choice of priors and Markov chain Monte Carlo settings
☒	☐	For hierarchical and complex designs, identification of the appropriate level for tests and full reporting of outcomes
☒	☐	Estimates of effect sizes (e.g. Cohen's d, Pearson's r), indicating how they were calculated

Our web collection on statistics for biologists contains articles on many of the points above.

Software and code

Policy information about availability of computer code

Data collection	Flow cytometry data was acquired using the BD FACSDIVA software, Kaluza software for Gallios (Beckman Coulter) or Attune NxT software. Microscopy data was collected using the ZEISS ZEN software. For absorbance measurements the Bio-Rad Microplate Manager 6 Software or the MARS Software from BMG Labtech was used. For collection of ESI-MS/MS data the Micromass MassLynx MS Software was used, for UPLC-ESI-HRMS/MS Bruker Compass DataAnalysis Version 4.3 was used and uHPLC data was collected using the Chromeleon 7.2 Chromatography Data System. TEER measurements was performed using a cellZscope system (nanoAnalytics GmbH).
Data analysis	Flow cytometry data was analyzed using FlowJo version 8 or X, or Kaluza v2.1.<br>Quantification of microscopy images was done using ImageJ.<br>Metabolomics analysis with ESI-MS/MS data was done with the Micromass MassLynx MS Software. For UPLC-ESI-HRMS/MS data Bruker TargetAnalysis Version 4.3 was used to determine Trp concentrations. For uHPLC the Chromeleon 7.2 Chromatography Data System was used for analysis of D-Trp levels.<br>TEER data was analyzed using the cellZscope v2.2.2 software.<br>Microbiota data was analyzed using an in-house shell script pipeline based on standard procedures for 16S rRNA gene sequence data. For further details please refer to the online methods sections. Scripts are available on reasonable request.<br>The functional potential of microbial communities was infered using PICRUSt. See respective methods section for details.<br>RNA-seq data was analyzed using R and included the use of the following packages: Trim Galore (version 0.4.4), Cutadapt and FastQC for trimming and QC; STAR aligner (version 2.5.2b) for mapping; featureCounts (version 1.5.2) for estimation of expression counts; and DESeq2 (version 1.20.0) for differential expression analysis. GO enrichment analysis was done using the topGO (version 2.32.0) package. For TFBS analysis innateDB (version 5.4) was used. Identification of interaction partners was done using BioGRID database (version 3.5). For network analysis the STRING database (version 10.0) was used. The full codes of all R scripts are available on reasonable request.<br>All other statistics were done using GraphPad prism 6.0.

For manuscripts utilizing custom algorithms or software that are central to the research but not yet described in published literature, software must be made available to editors/reviewers. We strongly encourage code deposition in a community repository (e.g. GitHub). See the Nature Research guidelines for submitting code & software for further information.

Data

Policy information about availability of data

All manuscripts must include a data availability statement. This statement should provide the following information, where applicable:

- Accession codes, unique identifiers, or web links for publicly available datasets
- A list of figures that have associated raw data
- A description of any restrictions on data availability

RNA-seq data that support the findings of this study has been deposited in the Gene Expression Omnibus repository and will be made available prior to publication. 16S rRNA gene sequencing data has been deposited in the European Nucelotide Archive and will be made available prior to publication. All additional data sets generated or analysed during this study are included in this published article (and its Supplementary Data and Source Data files).

Field-specific reporting

Please select the one below that is the best fit for your research. If you are not sure, read the appropriate sections before making your selection.

☒ Life sciences ☐ Behavioural & social sciences ☐ Ecological, evolutionary & environmental sciences

For a reference copy of the document with all sections, see nature.com/documents/nr-reporting-summary-flat.pdf

Life sciences study design

All studies must disclose on these points even when the disclosure is negative.

Sample size	Sample size was calculated with the help of a biostatistician using R version 3.4.0. Assumptions for power analysis were as follows: alpha error: 5%; beta error: 20%. Values for standard deviation and differences between experimental groups were based on previous experiments (whenever a similar data type was available). In all other cases a pilot group size was used.
Data exclusions	One colon sample was excluded from RNA-seq analysis since this sample failed the QC. Two additional RNA-seq colon samples were excluded due to contamination with rectal tissue.<br>One T cell sample was excluded from NanoString analysis due to low RNA quality.<br>Individual mice were removed from histology-based scoring analyses (e.g. immune infiltration) in case the tissue showed massive signs of destruction due to the tissue processing pipeline.<br>In case animals had to be sacrificed prior to the pre-defined endpoint (due to weight loss or other termination criteria), they were excluded from any downstream analysis.
Replication	Key experiments (EAE score dietary trp/protein depletion) were all performed at least twice and data from one representative experiment are shown in this manuscript. All other experiments were performed once with biological replicates or technical replicates (as specified in figure legend).
Randomization	Mice were matched into the groups according to age, sex and genotype (where applicable) at the time of treatment start.
Blinding	For EAE scoring and general health monitoring blinding was not feasible since animals have to be kept in separate cages due to the diet (which is color-coded). For histology-based analyses the experimentator was blinded when scoring e.g. demyelination, gut inflammation. For flow cytometry analysis gates were identified based on FMO controls and all samples were analyzed using the same gates.

Reporting for specific materials, systems and methods

We require information from authors about some types of materials, experimental systems and methods used in many studies. Here, indicate whether each material, system or method listed is relevant to your study. If you are not sure if a list item applies to your research, read the appropriate section before selecting a response.

Materials & experimental systems

n/a	Involved in the study	
☐	☒	Antibodies
☒	☐	Eukaryotic cell lines
☒	☐	Palaeontology
☐	☒	Animals and other organisms
☒	☐	Human research participants
☒	☐	Clinical data

Methods

n/a	Involved in the study	
☒	☐	ChIP-seq
☐	☒	Flow cytometry
☒	☐	MRI-based neuroimaging

Antibodies

Antibodies used	Conjugate Antigen, clone, distributor, catalogue number, LOT number

Purified anti-IFNg, XMG1.2, eBioscience, 16-7311-85, LOT: 4274994; 4316705
Biotinylated anti-CD8a, 53-6.7, eBioscience, 13-0081-85, LOT: E02386-1632; 4341608
Biotinylated anti-CD19, 6D5, BioLegend, 115504, LOT: B192314
Biotinylated anti-CD49b, DX5, BioLegend, 108904, LOT: B173249
Biotinylated anti-CD11c, N418, BioLegend, 117304, LOT: B239867
Biotinylated anti-CD11b, M1/70, BioLegend, 101204, LOT: B161661; B199301
Biotinylated anti-Ter119, Ter119, eBioscience, 13-5921-82, LOT: 4300555
Purified anti-CD3, polyclonal, Dako, A0452, LOT: 00081639; 20049827
Purified anti-CD107b, M3/84, BioLegend, 108502, LOT: B140336
Goat anti-rat IgG (H+L), Vector Laboratories, BA-9400, LOT: Y0731
Goat anti-rabbit IgG (H+L), Vector Laboratories, BA-1000, LOT: ZA0520; ZC0908
PE-Cy7 CCR6, 29-2L17, BioLegend, 129815, LOT: B252582
APC CD3, 17A2, BioLegend, 100236, LOT: B256436
Brilliant Violet CD3, 17A2, BioLegend, 100241, LOT: B265870
eFluor450 CD3, 17A2, eBioscience, 48-0032-82, LOT: 4314440
FITC CD3, 17A2, BioLegend, 100204
APC CD4, RM4-5, BioLegend, 100516, LOT: B218256; B243409
eFluor506 CD4, RM4-5, eBioscience; 69-0042-82, LOT: 4306078
FITC CD4, GK1.5, eBioscience, 11-0041-85, LOT: E00079-1632
Pacific Blue CD4, RM4-5, BioLegend, 100531, LOT: B255834
PerCP-Cy5.5 CD8a, 53-6.7, eBioscience, 45-0081-82, LOT: 4291993; 1941169
Brilliant Violet 421 CD44, IM7, BioLegend, 103047, LOT: B263658
BV510 CD45, 30-F11, BioLegend, 103137, LOT: B260403; B240739
PE-Cy7 CD45.1, A20, BioLegend, 100531, LOT: B233222
PE CD45.2, 104, BioLegend, 109808, LOT: B209478
FITC CXCR3, CXCR3-173, eBioscience, 11-1831-80, LOT: 1943417
PerCP-eFluor710 CD40L, MR1, eBioscience, 46-1541-82, LOT: 4280452
FITC FoxP3, FJK-16s, eBioscience, 11-5773-82, LOT: 4340671; 4276015
PerCP-Cy5.5 GM-CSF, MP1-22E9, BioLegend, 505410, LOT: B231050
FITC IFNg, XMG1.2, eBioscience, 11-7311-82, LOT: 4286058
PE IFNg, XMG1.2, eBioscience, 12-7311-82, LOT: 4278600; 4278606
PE IL10, JES5-16E3, eBioscience, 12-7101-82, LOT: E02095-1634
PE-Cy7 IL-17A, eBio17B7, eBioscience, 25-7177-82, LOT: 4300417
IgG1-HRP, polyclonal, Bethyl, A90-205P, LOT: A90-205P-3
IgM-HRP, polyclonal, Sigma-Aldrich, A8786
Streptavidin FITC, BioLegend, 405201, LOT: B154215

Validation

Magnetic sorting and in vitro stimulation antibodies have been titrated and established in previous experiments.
Validation data of flow cytometry and immunohistochemistry antibodies can be found on the suppliers' website, e.g. in technical data sheets.

Animals and other organisms

Policy information about studies involving animals; ARRIVE guidelines recommended for reporting animal research

Laboratory animals

C57BL/6J wild-type (WT) mice were purchased from Charles River or Janvier Labs.
GCN2 knockout (Gcn2−/−; B6.129S6-Eif2ak4tm1.2Dron/J), AHR knockout (Ahr-/-; B6.129-Ahrtm1Bra/J) and 2D2 (C57BL/6-Tg(Tcra2D2,Tcrb2D2)1Kuch/J) mice were ordered from Jax® Mice, The Jackson Laboratory and bred at the animal facility of the DKFZ Heidelberg.
C57BL/6J Ly5.1 mice (B6.SJL-Ptprca Pepcb/BoyJ) were provided by the Center for Preclinical Research of the DKFZ Heidelberg.
2D2 x Ly5.1 mice were generated in-house by crossing 2D2 mice to C57BL/6J Ly5.1 mice. Germ-free (GF) C57BL/6J mice were a kind gift of Christoph Reinhardt (Translational Research of Thrombosis & Hemostasis, Mainz).
Sex-, body weight- and age-matched mice were used for further experiments. If not stated otherwise, female mice were used for the experiments. All mice were 6-14 weeks of age at use. Mice were kept under SPF conditions at the animal facility of the DKFZ Heidelberg or in the GF facility in the TARC, University Medical Center Mainz.

Wild animals

n/a

Field-collected samples

n/a

Ethics oversight

Animal experiments were performed according to the rules of the German Animal Welfare Act and were licensed by the regional authority Karlsruhe. This study did not involve any human material.

Note that full information on the approval of the study protocol must also be provided in the manuscript.

Flow Cytometry

Plots

Confirm that:

☒ The axis labels state the marker and fluorochrome used (e.g. CD4-FITC).

☒ The axis scales are clearly visible. Include numbers along axes only for bottom left plot of group (a 'group' is an analysis of identical markers).

☒ All plots are contour plots with outliers or pseudocolor plots.

☒ A numerical value for number of cells or percentage (with statistics) is provided.

Methodology

Sample preparation	Single cell suspensions from spleen and dLN samples were generated by meshing tissue through a 70 µm cell strainer twice (2 washes with 20 ml PBS each). Erythrocytes were removed from spleen samples using ACK lysis. In some cases, as described in online methods, cells were stimulated with peptides and Brefeldin A before analysis. If required, single cell suspension were labeled with CellTrace Far Red according to the manufacturer's instructions (see online methods).<br>CNS-infiltrating leukocytes were obtained from spinal cord tissue by mechanical dissection and enzymatic digestion with Liberase DL as specified in the methods section.
Instrument	BD Canto II, ThermoFisher Attune NxT, Beckman Coulter Gallios, BD Aria II
Software	Flow cytometry data was acquired using the BD FACSDIVA Software. Data analysis was done using FlowJo 8 or X.
Cell population abundance	Flow cytometry-activated cell sorting for NanoString analysis: Approx. 1.5% of total cells
Gating strategy	Lymphocytes were defined by size and granularity in FSC-A vs. SSC-A plots. Subsequently, duplets were excluded in FSC-W vs FSC-H plots and dead cells were excluded by means of fixable viability dye positivity. T cells were gated according to the lineage marker CD3. CD4+ T cells and CD45.2+ T cells were gated in CD4 or CD45.2 vs. CD3 plots.<br>Boundaries between positive and negative cells for intracellular markers (IFNg, FoxP3, IL17A) were defined by use of fluorescence minus one (FMO) controls. Increase in chemokine receptor expression was determined by calculating the median fluorescence intensity (MFI).<br>For flow cytometry activated cell sorting dead cells were exlcuded by means of fixable viability dye positivity. Remaining biotin-positive cells (e.g. B cells, NK cells, monocytes) were removed by streptavidin FITC staining. FITC- CD3+ CD4+ CD45.1+ cells were sorted as cells which are positive for the transgenic MOG35-55-reactive TCR.

☒ Tick this box to confirm that a figure exemplifying the gating strategy is provided in the Supplementary Information.
